# ORP8 acts as a lipophagy receptor to mediate lipid droplet turnover

**DOI:** 10.1093/procel/pwac063

**Published:** 2022-12-07

**Authors:** Maomao Pu, Wenhui Zheng, Hongtao Zhang, Wei Wan, Chao Peng, Xuebo Chen, Xinchang Liu, Zizhen Xu, Tianhua Zhou, Qiming Sun, Dante Neculai, Wei Liu

**Affiliations:** Metabolic Medicine Center, International Institutes of Medicine, the Fourth Affiliated Hospital, Zhejiang University School of Medicine, Yiwu 322000, China; Metabolic Medicine Center, International Institutes of Medicine, the Fourth Affiliated Hospital, Zhejiang University School of Medicine, Yiwu 322000, China; Metabolic Medicine Center, International Institutes of Medicine, the Fourth Affiliated Hospital, Zhejiang University School of Medicine, Yiwu 322000, China; Metabolic Medicine Center, International Institutes of Medicine, the Fourth Affiliated Hospital, Zhejiang University School of Medicine, Yiwu 322000, China; National Center for Protein Science Shanghai, Institute of Biochemistry and Cell Biology, Shanghai Institutes of Biological Sciences, Chinese Academy of Sciences, Shanghai 200031, China; Metabolic Medicine Center, International Institutes of Medicine, the Fourth Affiliated Hospital, Zhejiang University School of Medicine, Yiwu 322000, China; Metabolic Medicine Center, International Institutes of Medicine, the Fourth Affiliated Hospital, Zhejiang University School of Medicine, Yiwu 322000, China; Metabolic Medicine Center, International Institutes of Medicine, the Fourth Affiliated Hospital, Zhejiang University School of Medicine, Yiwu 322000, China; Metabolic Medicine Center, International Institutes of Medicine, the Fourth Affiliated Hospital, Zhejiang University School of Medicine, Yiwu 322000, China; Metabolic Medicine Center, International Institutes of Medicine, the Fourth Affiliated Hospital, Zhejiang University School of Medicine, Yiwu 322000, China; Metabolic Medicine Center, International Institutes of Medicine, the Fourth Affiliated Hospital, Zhejiang University School of Medicine, Yiwu 322000, China; Metabolic Medicine Center, International Institutes of Medicine, the Fourth Affiliated Hospital, Zhejiang University School of Medicine, Yiwu 322000, China; Joint Institute of Genetics and Genomics Medicine between Zhejiang University and University of Toronto, Hangzhou 310058, China

**Keywords:** ORP8, lipophagy, lipid, autophagy

## Abstract

Lipophagy, the selective engulfment of lipid droplets (LDs) by autophagosomes for lysosomal degradation, is critical to lipid and energy homeostasis. Here we show that the lipid transfer protein ORP8 is located on LDs and mediates the encapsulation of LDs by autophagosomal membranes. This function of ORP8 is independent of its lipid transporter activity and is achieved through direct interaction with phagophore-anchored LC3/GABARAPs. Upon lipophagy induction, ORP8 has increased localization on LDs and is phosphorylated by AMPK, thereby enhancing its affinity for LC3/GABARAPs. Deletion of ORP8 or interruption of ORP8-LC3/GABARAP interaction results in accumulation of LDs and increased intracellular triglyceride. Overexpression of ORP8 alleviates LD and triglyceride deposition in the liver of *ob/ob* mice, and *Osbpl8*^*-/-*^ mice exhibit liver lipid clearance defects. Our results suggest that ORP8 is a lipophagy receptor that plays a key role in cellular lipid metabolism.

## Introduction

Autophagy is a lysosome-dependent cell degradation pathway that functions not only to meet the nutritional and energy needs, but also as a key mediator of cell homeostasis. The latter, to a large extent, is achieved by selectively eliminating damaged or unwanted organelles and deleterious protein aggregates (Levine and Kroemer, 2019). There is increasing evidence that selective autophagy constitutes the basic mechanism of the quality and quantity control of organelles, and participates in the regulation of cell growth, metabolism and fate determination (Novak et al., 2010; Gump et al., 2014; Khaminets et al., 2015; Wyant et al., 2018).

Lipid droplets (LDs) are unique intracellular organelles that are filled with neutral lipids and surrounded by phospholipid monolayers. As highly dynamic structures, LDs repeat the cycle of formation and degradation. As for the degradation of LDs, in addition to the decomposition of triglycerides (TG) in LDs through lipolysis (Zechner et al., 2017), LDs can also be wrapped by autophagosomes for lysosomal degradation, that is, lipophagy. It has been shown that LDs can interact with autophagy machinery, and inhibition of autophagy leads to the increase of LD number and size and TG level in cells (Singh et al., 2009). In addition to hepatocytes and adipocytes containing high levels of lipids (Singh et al., 2009; Martinez-Lopez et al., 2016), lipophagy has also been identified in neurons (Kaushik et al., 2011), macrophages (Ouimet et al., 2011) and fibroblasts (Kaushik and Cuervo, 2015). Recent studies revealed that lipophagy is related to neuroinflammation (Xu et al., 2021), thermogenesis (Martinez-Lopez et al., 2016), and aging (O’Rourke and Ruvkun, 2013). Lack of lipophagy has been associated with metabolic disorders, such as liver steatosis (Settembre et al., 2013; Lin et al., 2016; Tanaka et al., 2016; Zubiete-Franco et al., 2016).

Selective engulfment of organelles or protein aggregates by autophagosomes requires phagophore-associated LC3/GABARAP proteins to interact directly with specific organelle receptors, or to bind to adaptor proteins that are organelle-associated (Levine and Kroemer, 2019). So far, many such receptors have been found to guide the encapsulation of mitochondria (Novak et al., 2010; Liu et al., 2012; Wei et al., 2017), endoplasmic reticulum (ER) (Khaminets et al., 2015; Fumagalli et al., 2016; Chino et al., 2019) and ribosomes (Wyant et al., 2018). However, the proteins that specifically mediate the recognition and envelopment of LDs remain to be determined, although several LD-associated proteins may be involved (Kaushik and Cuervo, 2015; Schroeder et al., 2015; Li et al., 2016; Garcia-Macia et al., 2021). Here, by screening LD proteins that potentially interact with LC3, we have identified the key role of oxysterol-binding protein (OSBP)-related protein 8 (ORP8), a known ER lipid transfer protein, in lipophagy. Our data suggest that ORP8 acts as a specific lipophagy receptor to mediate LD turnover.

## Results

### ORP8 is localized on LDs

In the search for LD-localized proteins that may mediate lipophagy, we purified LDs from HeLa cells treated with oleic acid (OA), which stimulates LD biogenesis and lipophagy (Singh et al., 2009), together with chloroquine (CQ), which inhibits lysosomal degradation (Mauthe et al., 2018). We incubated the LD lysates with purified GST-LC3B, and then pulled down GST-LC3B for mass spectrometry analysis (Fig. S1A). Among the proteins pulled down with LC3B (Table S1) was the ER-localized lipid transporter ORP8, which was previously found to be associated with LC3 by affinity proteomics (Behrends et al., 2010). To verify the interaction between ORP8 and LC3, we performed co-precipitation assays in HeLa cells under serum starvation which induces lipophagy more strongly than non-selective autophagy (Rambold et al., 2015). Immunoprecipitation of LC3B co-precipitated ORP8, but not the other OSBP family members including OSBP, ORP5 or ORP2 (Fig. S1B). These data therefore indicate a specific association of ORP8 with LC3 and a possible involvement of ORP8 in lipophagy.

**Figure 1.**
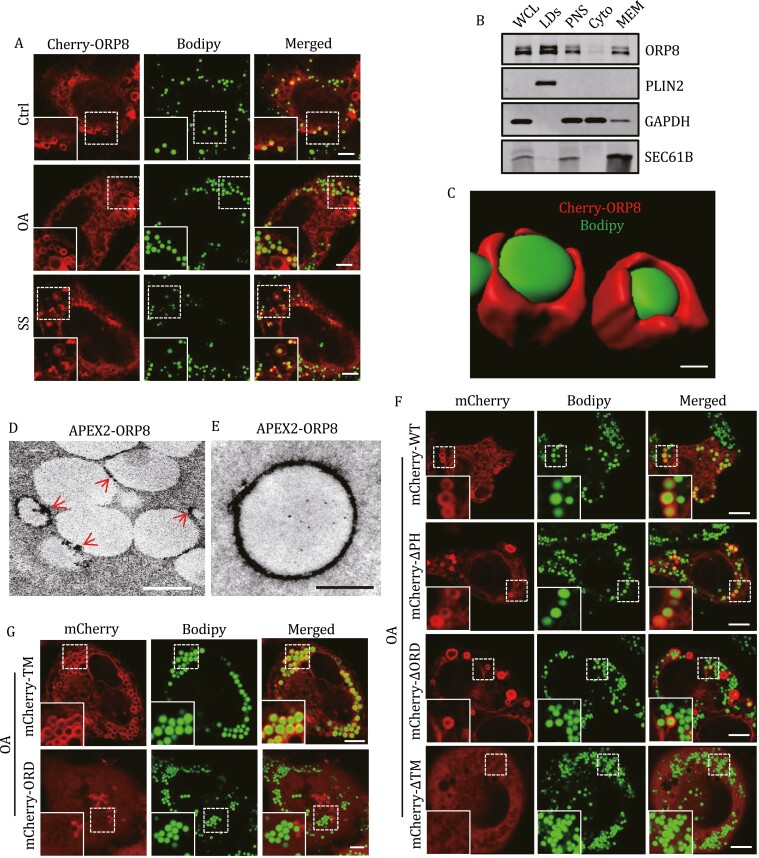
**The localization of ORP8 on LDs.** (A) Live-cell images of mCherry-ORP8-expressing HeLa cells treated with 200 µmol/L OA or serum-starved for 6 h. Cells were stained with Bodipy. (B) Endogenous ORP8 and PLIN2 proteins in the subcellular fractions of HeLa cells were analyzed by Western blot. Cells were treated with 200 µmol/L OA for 12 h. WCL, whole cell lysate; PNS, post-nuclear supernatant; Cyto, cytosol; MEM, cellular membranes. (C) 3D reconstruction image of LDs coated with mCherry-ORP8. (D) APEX-EM image of HeLa cells expressing HA-APEX2-ORP8. The cells were treated with 200 µmol/L OA for 6 h. The arrow indicates APEX2-ORP8 signal on LDs. (E) APEX-EM image of the HA-APEX2-ORP8 on the surface of a purified LD. LDs were purified from HeLa cells expressing HA-APEX2-ORP8 which were pre-treated with 200 µmol/L OA for 12 h. Purified LDs were embedded in 6% gelatin, and performed the APEX-EM analysis. (F and G) Confocal images of HeLa cells expressing the indicated mCherry-ORP8 truncated mutants. Cells were treated with 200 µmol/L OA for 6 h. OA, oleate acid; SS, serum starvation. Scale bars, 5 µm (A, F, G); 0.5 µm (C–E).

We then sought to clarify the localization of ORP8 on LDs. In HeLa cells under basal culture conditions, a small portion of mCherry-ORP8 presented punctate or ring-like signals and co-localized to Bodipy-stained LDs (Fig. 1A). Co-localization of mCherry-tagged ORP8 to Bodipy-stained LDs was also observed in HEK293 cells and Huh7 cells (Fig. S1C). mCherry-ORP8 puncta did not overlap with the integral ER membrane protein Sec61, which ruled out the possibility of mCherry-ORP8 accumulation on ER membranes (Fig. S1D). OA treatment or serum starvation increased the co-localization of mCherry-ORP8 and Bodipy (Figs. 1A and S1E). By contrast, the overlap of Bodipy with mCherry-tagged ORP5, another ORP family member with a similar domain structure to ORP8, was only detected in OA-treated (Du et al., 2020) but not untreated or serum-starved cells (Fig. S1F). We then purified LDs from OA-treated cells (Fig. S1G), and Western blot analysis confirmed that a proportion of endogenous ORP8 was LD-associated (Figs. 1B and S1H). Due to the lack of effective ORP8 antibody for immunostaining, we inserted mCherry into endogenous ORP8 gene (*OSBPL8*) using CRISPR-Cas9 system. Obviously, in OA-treated cells, endogenous mCherry-ORP8 was co-localized with Bodipy-labeled LDs (Fig. S1I). Moreover, three-dimensional reconstruction of Airyscan confocal images and APEX-electron microscopy (APEX-EM) also showed the distribution of ORP8 on the surface of LDs in HeLa cells (Fig. 1C and 1D). We further analyze the LDs purified from HeLa cells expressing APEX2-ORP8 by APEX-EM. The results showed that APEX2-ORP8 was located on the surface of separated LDs without other membranes (Fig. 1E).

To characterize the LD targeting of ORP8, we constructed different ORP8 truncated mutants and expressed them in cells. mCherry-tagged ORP8 lacking the N-terminal PH domain or the conserved ORD domain still located on LDs, while ORP8 without the C-terminal TM domain was fully dispersed in the cytoplasm (Figs. 1F and S1J). Consistent with this, when most ORP8 containing only the TM domain was on the surface of LDs, ORP8 containing only the ORD domain was completely separated from LDs (Figs. 1G and S1J). These observations suggest that, unlike ORP5, which uses its ORD domain to associate LDs (Du et al., 2020), ORP8 may translocate to LDs through its TM domain. In fact, we have produced an ORP5 mutant whose TM domain is replaced by the TM domain of ORP8. We found that this ORP5 mutant can indeed localize to LDs even in cells without OA treatment (Fig. S1K). Taken together, these data confirmed the LD-localization of ORP8, which is increased under conditions that induce lipophagy.

### ORP8 promotes LD degradation

**Figure 2.**
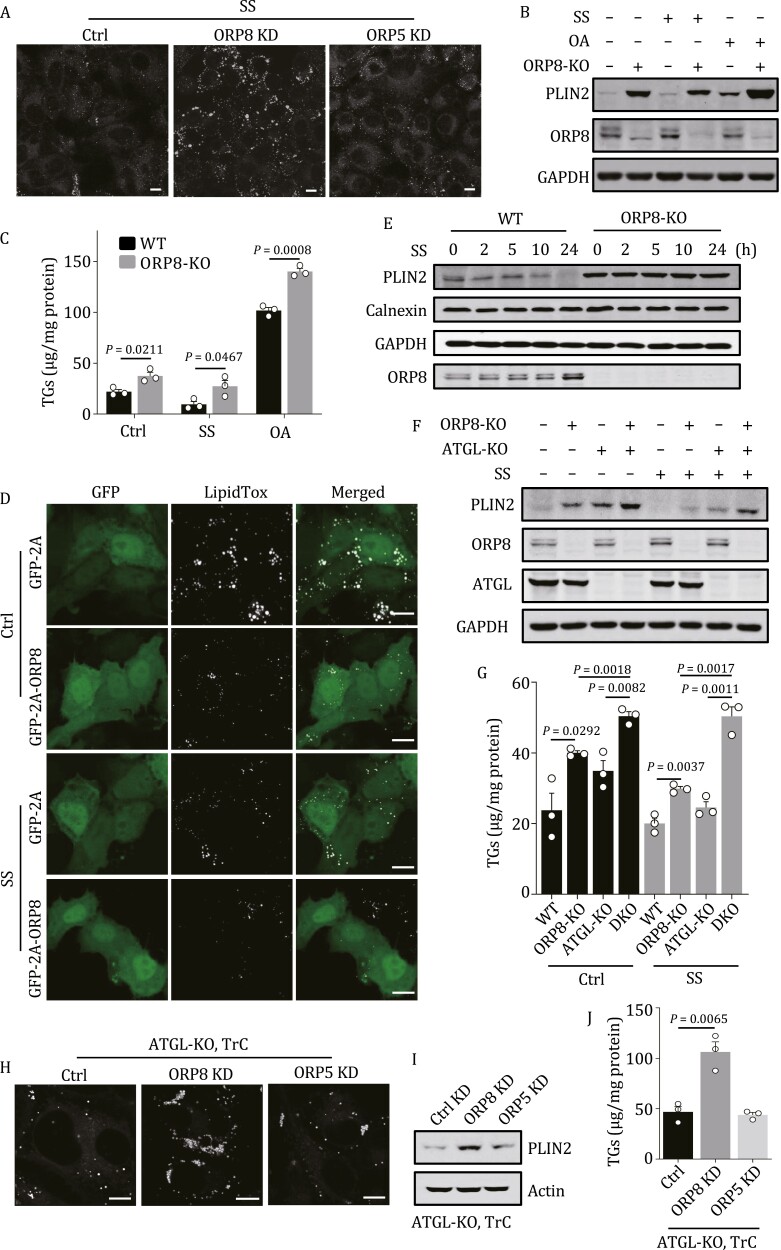
**ORP8 promotes turnover of LDs.** (A) Maximum intensity projection (MIP) confocal images of HeLa cells treated with ORP8 or ORP5 siRNA for 48 h and serum starvation for 24 h. The cells were stained with Bodipy. (B) PLIN2 in WT and ORP8-KO HeLa cells treated with or without serum starvation or 200 µmol/L OA for 24 h was analyzed by Western blot. (C) TG level in cells treated with or without serum starvation or 200 µmol/L OA for 24 h. Quantitative data are derived from three independent experiments. (D) MIP images of HeLa cells expressing GFP-2A or GFP-2A-ORP8 with or without serum starvation for 24 h. The cells were stained with LipidTox. (E) WT or ORP8-KO HeLa cells were pre-treated with 200 µmol/L OA overnight and cultured in serum-free medium containing 6 μmol/L TrC for the indicated times. Cell lysates were then analyzed by Western blot using the indicated antibodies. (F and G) PLIN2 expression (F) and TG levels (G) in WT or the indicated ORP8 or/and ATGL-KO HeLa cells with or without serum starvation for 24 h. Results are reported as means ± SEM of three replicates (G). (H) Bodipy staining of ATGL-KO HeLa cells pre-loaded with 200 µmol/L OA overnight and cultured with 6 μmol/L TrC and ORP8 siRNA or ORP5 siRNA for 48 h. (I and J) PLIN2 expression (I) and TG levels (J) in ATGL-KO cells treated as (H). Quantitative data are derived from three independent experiments (J). OA, oleate acid; SS, serum starvation; TrC, triacsin C. Scale bars, 10 μm.

Overexpression of ORP8 reduced TGs in preadipocytes (Zhou et al., 2012) and in mouse liver and plasma (Zhou et al., 2011). Thus, we assessed the potential role of ORP8 in LD metabolism. Knockdown (KD) of ORP8 but not ORP5 significantly increased the LD content of serum-starved cells (Figs. 2A and S2A–C). We created an ORP8 knockout (KO) HeLa cell line using CRISPR-Cas9 system. Deletion of ORP8 caused dramatic accumulation of perilipin2 (PLIN2), a LD-binding protein most commonly used as a LD marker, in KO cells with or without OA treatment or serum starvation (Fig. 2B). The level of TG was also increased in these cells (Fig. 2C). These results were confirmed in ORP8-KO HEK293 cells (Fig. S2D and S2E). We next evaluated the effect of ORP8 overexpression by transfecting a GFP-2A-OPR8 plasmid, in which self-cleaving peptide T2A was inserted between GFP and OPR8 (Szymczak et al., 2004). This allows GFP and ORP8 to be expressed separately at a comparable level to rule out the potential impact of GFP on ORP8 function. Under either basal or serum starvation conditions, expression of GFP-2A-OPR8 significantly decreased cellular LD content (Figs. 2D and S2F), while overexpression of HA-ORP8 but not HA-ORP5, significantly reduced TG level in the cells (Fig. S2G). Taken together, these results suggest that ORP8 negatively regulates LD metabolism.

To prove that ORP8 functions in LD catabolism, we blocked LD biogenesis and then analyzed the consumption of LDs in ORP8-KO cells. The cells were pre-treated with OA and then transferred to serum-free medium containing triacsin C (TrC), a specific inhibitor of fatty acyl CoA synthetase. Under serum starvation conditions, PLIN2 protein was reduced in TrC-treated WT cells, but not in TrC-treated ORP8-KO cells (Fig. 2E). In addition, while KO of adipose triglyceride lipase (ATGL), the rate-limiting lipase on LDs, increased intracellular PLIN2 and TG levels, double KO of ATGL and ORP8 caused further accumulation of PLIN2 and TG (Fig. 2F and 2G). These results indicated that ORP8 promoted LD degradation independent of lipolysis. This conclusion was confirmed by knocking down ORP8 but not ORP5 in TrC-treated ATGL-deleted cells in which both LD synthesis and lipolysis were blocked (Figs. 2H–J, S2H and S2I).

ORP8 and ORP5 are well-known lipid transporters which transfer phosphoserine (PS) from ER to plasma membrane and phosphatidylinositol 4-phosphate (PI4P) from plasma membrane to ER (Chung et al., 2015). Recently, ORP5 was also shown to transport PI4P and PS between ER and LDs (Du et al., 2020). We therefore observed the effect of a lipid transport-inactive ORP8 mutant, ORP8-H514A/H515A (Chung et al., 2015). Strikingly, like WT ORP8, overexpression of GFP-2A-ORP8-H514A/H515A significantly reduced LDs in cells (Fig. S2J–L). These data suggest that the involvement of ORP8 in LD degradation may not be related to its lipid transport activity.

### ORP8 regulates lipophagy

**Figure 3.**
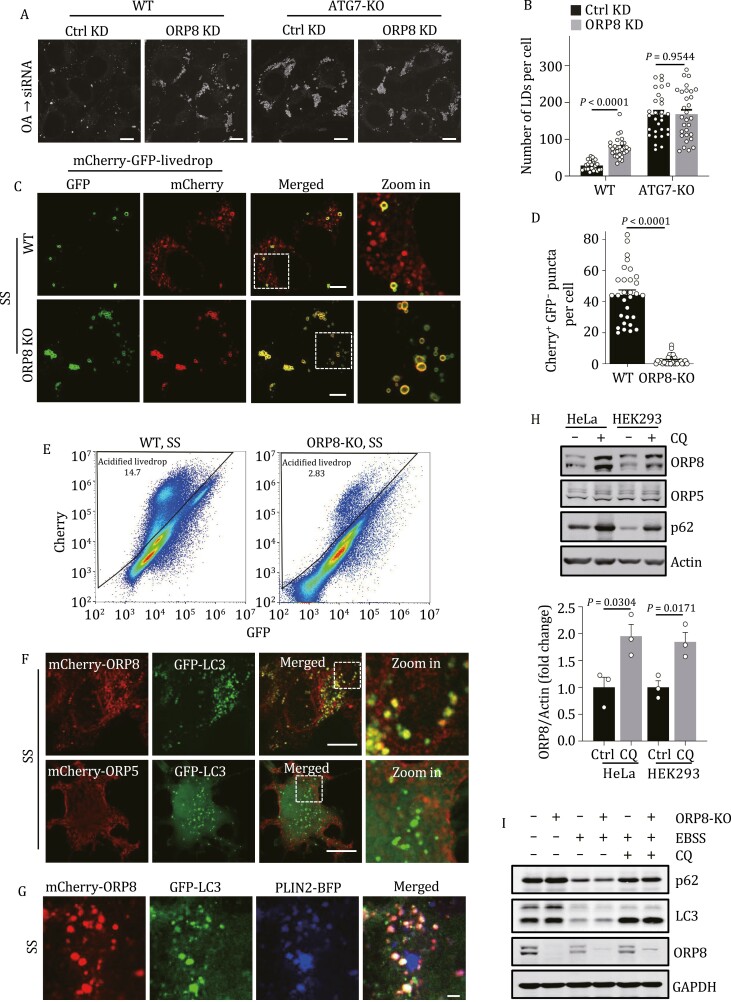
**ORP8 is required for lipophagy.** (A) WT and ATG7-KO MEFs stained with Bodipy. Cells were pre-loaded with OA overnight and treated with or without ORP8 siRNA for 48 h in regular medium. (B) Quantification of LD number per cell in (A). *n* = 30 cells. (C) WT or ORP8-KO HeLa cells stably expressing mCherry-GFP-livedrop were serum-starved for 24 h and imaged by confocal microscopy. (D) Quantification of the number of mCherry^+^ GFP^-^ puncta per cell in (C). *n* = 30 cells. (E) Representative FACS scatterplots of GFP and mCherry fluorescence in WT or ORP8-KO cells. Cells expressing mCherry-GFP-livedrop were serum-starved for 24 h. (F) Confocal images of GFP-LC3-expressing HeLa cells transfected with mCherry-ORP8 or mCherry-ORP5. The cells were serum-starved for 24 h. (G) Co-localization of mCherry-ORP8, GFP-LC3 and PLIN2-BFP in serum-starved HeLa cells for 24 h. (H) The indicated proteins in HeLa cells and HEK293 cells with or without 50 μmol/L CQ treatment for 24 h were analyzed by Western blot (upper panel). Quantification of the relative protein level of ORP8 (lower panel). Quantitative data are from three independent experiments. (I) The indicated proteins in WT and ORP8-KO HeLa cells cultured in Earle’s balanced salt solution (EBSS) for 12 h with or without 50 μmol/L CQ for 6 h were analyzed by Western blot. OA, oleate acid; SS, serum starvation; CQ, chloroquine. Scale bars, 10 μm (A, C, F), 2 μm (G).

We then investigated whether ORP8 regulates LDs through lipophagy. ORP8 KD significantly increased the number of LDs in WT MEFs, but not in MEFs lacking ATG7 (Figs. 3A, 3B and S3A). This suggests that the function of ORP8 depends on the core autophagy machinery. We then constructed a probe in which mCherry and GFP were tandem-tagged to livedrop, a specific marker for LDs (Wang et al., 2016). In mCherry-GFP-livedrop expressing WT cells, serum starvation stimulated the production of red puncta (mCherry^+^ and GFP^−^). This corresponds to the quenching of acidity-sensitive GFP in lysosomes, indicating enhanced lipophagy flux. By contrast, most of the livedrop puncta remained yellow (mCherry^+^ and GFP^+^) in ORP8-KO cells (Fig. 3C and 3D). In accordance with this, fluorescence-activated cell sorting (FACS) of the ORP8-KO cells detected significantly fewer cells in the mCherry > GFP gate (Fig. 3E). We also performed pulse-chase assays by adding Bodipy Red C12, a fatty acid analog, to cultured cells expressing GFP-LC3. After one night pulse, Red C12 was incorporated into LDs in WT and ORP8-KO cells. However, while serum starvation resulted in the co-localization of Red C12 and GFP-LC3 in WT cells, this co-localization was rarely detectable in ORP8-KO cells (Fig. S3B and S3C). Electron microscopy analysis also showed that there was fewer autophagosomes containing lipids in ORP8-KO cells when compared with WT cells (Fig. S3D and S3E).

Consistent with these results, in serum-starved cells, LDs labeled with mCherry-ORP8 but not mCherry-ORP5 were co-localized with GFP-LC3 puncta (Fig. 3F and 3G) and CFP-LAMP1-labeled lysosomes (Fig. S3F). Finally, in CQ-treated cells, we detected the accumulation of endogenous ORP8 but not ORP5 (Fig. 3H). Taken together, these results indicated that ORP8 plays a crucial role in lipophagy by targeting LDs. These observations were not due to a possible effect of ORP8 on non-selective autophagy, because ORP8 deletion affected neither p62 protein level (Fig. 3I) nor autophagosome formation (Fig. S3G and S3H) in cells starved in Earle’s balanced salt solution (EBSS) or treated with CQ.

### ORP8 acts as a lipophagy receptor

**Figure 4.**
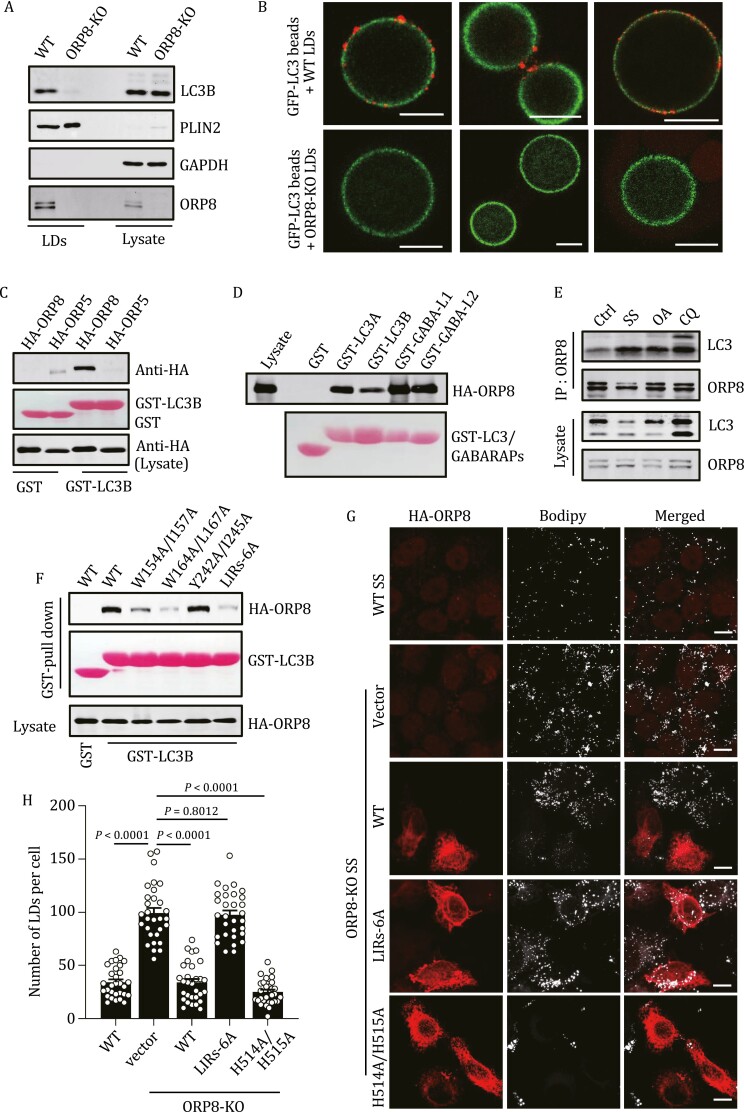
**Characterization of the interaction of ORP8-LC3.** (A) The indicated proteins in the subcellular fraction of HeLa cells were analyzed by Western blot. The cells were treated with 200 μmol/L OA for 12 h. (B) GFP-LC3 membranes from EBSS-treated HEK 293 cells stably expressing GFP-LC3 were pulled down by GFP-TRAP beads. Then the beads were incubated with equivalent Lipi-blue stained LDs purified from WT HeLa cells or ORP8-KO HeLa cells. The beads were then imaged by confocal microscope. Note the binding of LDs (red) from WT but not ORP8-KO cells to beads. In order to better show the attachment of LDs to the beads, we used red to mark Lipi-Blue. Scale bar, 10 μm. (C) Purified GST-LC3B was incubated with HEK293T cell lysates overexpressing HA-ORP8 or HA-ORP5, pulled down by GSH beads, and the bound HA-ORP8 and HA-ORP5 were analyzed by Western blot using anti-HA antibody. (D) Purified GST-tagged ATG8 family proteins were incubated with HEK293T cell lysates overexpressing HA-ORP8, pulled down by GSH beads, and the bound HA-ORP8 was analyzed by Western blot. (E) Co-immunoprecipitation of LC3B with ORP8 from HeLa cells. The cells were serum-starved for 24 h, or treated with 200 μmol/L OA or 50 μmol/L CQ for 6 h. (F) Purified GST-LC3B was incubated with HEK293T cell lysates overexpressing WT ORP8 or ORP8 mutants, pulled down by GSH beads, and the bound HA-ORP8 was analyzed by Western blot. (G) Representative MIP confocal images of WT and ORP8-KO cells with or without overexpression of HA-ORP8 WT, HA-ORP8-LIRs-6A or HA-ORP8-H514A/H515A. Cells were serum-starved for 24 h and stained by Bodipy. Scale bars, 10 μm. (H) Quantification of the number of LDs per cell in (G). *n* = 30 cells. OA, oleate acid; SS, serum starvation; CQ, chloroquine; GABA, GABARAP; LIR, LC3-interacting region.

The function of ORP8 in lipophagy and its interaction with LC3 strongly suggest that LD-localized ORP8 may serve as a receptor for targeting LDs to autophagosomes. This was supported by the data showing increased ORP8 in ATG7-KO cells (Fig. S3A) and cells treated with CQ (Fig. 3H). To verify this and clarify the functional mechanism of ORP8, we purified LDs from OA-treated cells. As expected, membrane-bound LC3-II was abundant on LDs from WT cells, but was eliminated from LDs from ORP8-KO cells (Fig. 4A). We then performed *in vitro* LD-membrane binding assays. GFP-LC3-positive membranes were pulled down from GFP-LC3-stable HEK293 cells using GFP-TRAP magnetic beads and incubated with purified LDs from WT or ORP8-KO HeLa cells. Obviously, GFP-LC3 beads recruited LDs from WT cells but not ORP8-KO cells (Figs. 4B and S4A).

To further characterize the interaction between ORP8 and LC3, we performed GST pull-down assays and found that purified recombinant GST-LC3B pulled down HA-ORP8, but not HA-ORP5, from lysates of transfected cells (Fig. 4C). Intriguingly, ORP8 also showed affinity with other tested Atg8 family members including LC3A, LC3B, GABARAPL1 and GABARAPL2 (Fig. 4D). In addition, co-immunoprecipitation analysis detected the interaction between endogenous ORP8 and LC3-II, which was stimulated by serum starvation or OA treatment (Fig. 4E). In contrast, endogenous ORP5 could not co-precipitate LC3 from cells treated with serum-starved or OA (Fig. S4B). Moreover, *in vitro* pull-down assays using purified recombinant ORP8 and LC3/GABARAP verified direct binding of ORP8 with each of the tested LC3 and GABARAP proteins (Fig. S4C). As many other LC3/GABRARAP-interacting proteins (Mizushima, 2020), the binding of ORP8 to GABARAPs was stronger than to LC3s.

Three potential LC3 interaction regions (LIRs) were predicted in ORP8 (Jacomin et al., 2016), including aa 154–157 and aa 164–167, which are completely conserved in vertebrates, and aa 242–245. To test the role of these LIRs, we produced ORP8 mutants in which each LIR was destroyed separately. GST-LC3 pull-down analysis showed that destroying the first (W154A/I157A) or the second (W164A/L167A) or all three LIRs (LIRs-6A), but not the third LIR (Y242/I245A), dramatically reduced the affinity of ORP8 for LC3B (Fig. 4F). In line with this, the ORP8-LIRs-6A mutant failed to co-localize with LC3 in serum-starved cells (Fig. S4D). In addition, overexpression of ORP8-WT or lipid transport-inactive ORP8-H514A/H515A, but not ORP8-LIRs-6A, significantly restored serum starvation-stimulated LD degradation in ORP8-deficient cells (Fig. 4G and 4H). Taken together, these results suggest that the direct interaction between LD-localized ORP8 and membrane-associated LC3/GABARAP is required for phagophores to target LDs.

Several adaptor proteins have been previously identified to mediate selective autophagy of organelles or invading pathogens (Levine and Kroemer, 2019). More recently, mass spectrometry analysis of LDs from macrophage foam cells suggested that three of these adaptors, SQSTM1/p62, NBR1 and OPTN, may be involved in autophagic degradation of LDs (Robichaud et al., 2021). We tested the possible participation of these adaptor proteins in ORP8-regulated lipophagy. In HeLa cells with NDP52/OPTN/TAX1BP1/NBR1/p62 deletion (Penta- KO) (Lazarou et al., 2015), serum starvation still reduced the level of PLIN2 (Fig. S4E) and the content of LDs (Fig. S4F). Moreover, in these cells, in the case of serum starvation, depletion of ORP8 but not ORP5 could still preserve LDs (Fig. S4F), and mCherry-ORP8 labeled LDs were still co-localized with GFP-LC3 (Fig. S4G). These data suggest that the known adaptor proteins are not involved in ORP8-mediated lipophagy.

### AMPK phosphorylates and activates ORP8

**Figure 5.**
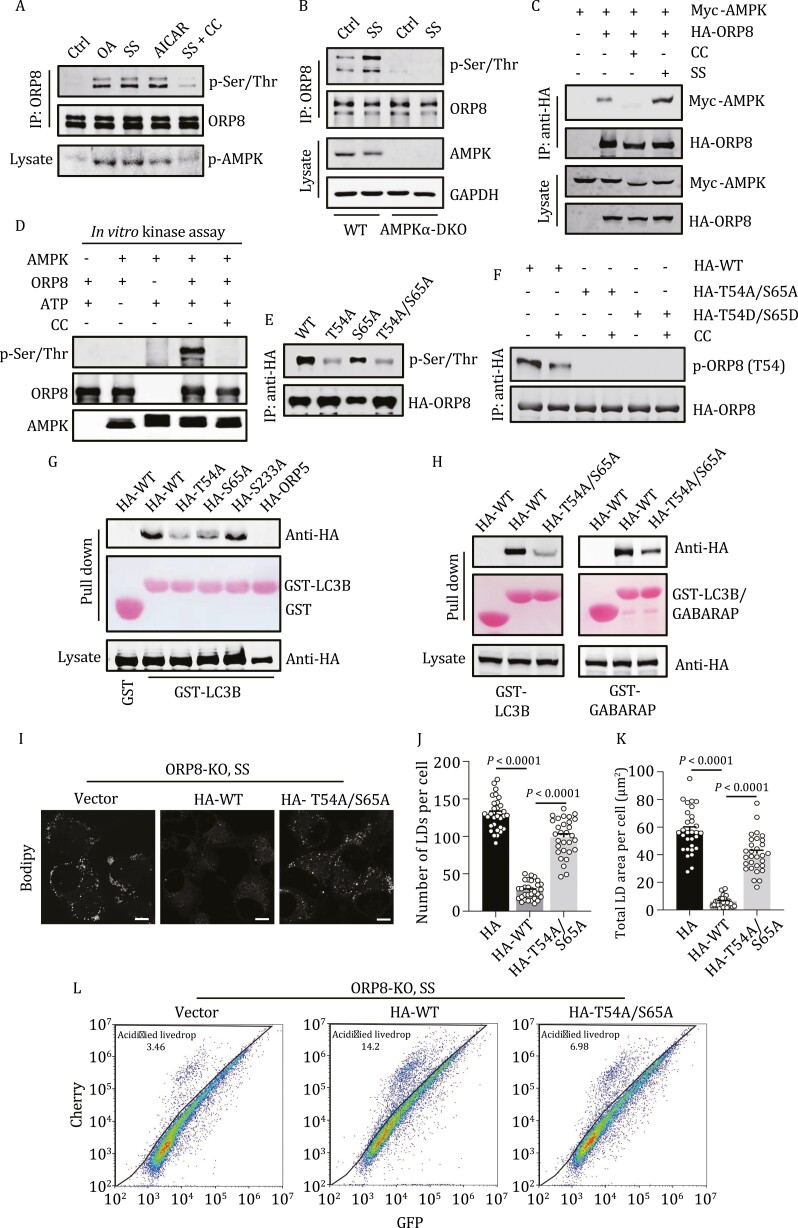
**AMPK regulates ORP8 activity in lipophagy.** (A) Phosphorylation of ORP8 in HeLa cells. Cells were treated with 200 μmol/L OA for 6 h or 2 mmol/L acadesine (AICAR) for 1 h, or were serum-starved for 24 h with or without 20 μmol/L compound C for 6 h. ORP8 was immunoprecipitated by anti-ORP8 and analyzed using an anti-phosphoserine/threonine antibody. (B) Phosphorylation of ORP8 in WT or AMPK α1/α2-DKO MEF cells. Cells were treated with or without serum starvation for 24 h. (C) Co-immunoprecipitation of Myc-AMPK with HA-ORP8 from transfected HEK293T cells. Cells were treated with or without 20 μmol/L compound C for 6 h or were serum- starved for 24 h. (D) *In vitro* kinase assay of AMPK using purified active AMPK complex (0.2 μg) with purified ORP8 (0.5 μg) as substrate. (E) Phosphorylation of ORP8 mutants in transfected HEK293T cells. Cells were glucose- and serum-starved for 6 h. Immunoprecipitated ORP8 was analyzed by Western blot using a pan phosphoserine/threonine antibody. (F) Phosphorylation of HA-tagged ORP8 and ORP8 mutants in HEK293T cells. Cells were glucose- and serum-starved for 6 h in the presence or absence of 20 μmol/L Compound C. HA-tagged ORP8 was immunoprecipitated with anti-HA and analyzed by Western blot using a specific antibody against ORP8 phosphorylated at Thr54. (G and H) Protein pull-down assay of ORP8 mutants and ORP5 from transfected HEK293T cell lysates after incubation with purified GST-LC3B (G and H) or GST-GABARAP (H). The cells were glucose- and serum-starved for 6 h. (I) Confocal images of ORP8-KO cells transfected with HA-tagged ORP8 or ORP8-T54A/S65A. The cells were serum-starved for 24 h and stained by Bodipy. (J and K) Quantification of the number (J) and total area (K) of LDs per cell in (I). *n* = 30 cells. Scale bar, 10 μm. (L) FACS scatterplots of GFP and mCherry fluorescence in mCherry-GFP-livedrop expressing ORP8-KO cells. Cells were transfected with or without HA-ORP8-WT or HA-ORP8-T54A/S65A and were serum-starved for 24 h. OA, oleate acid; CC, compound C; SS, serum starvation.

AMPK activates non-selective autophagy in response to energy deficiency. Previous studies have suggested the involvement of AMPK in lipophagy (Jordan and Randall, 2017; Zhang et al., 2020), which may be related to its role in initiating phagophore formation. In addition, it was recently revealed that CoA esters of long-chain fatty acids can allosterically activate the β1-containing AMPK that is predominantly expressed in hepatocytes (Pinkosky et al., 2020). Therefore, we investigated the potential role of AMPK in ORP8-mediated lipophagy in order to understand the regulatory mechanism. First, we confirmed that both OA treatment and serum starvation activated AMPK in HeLa cells (Figs. 5A and S5A). Intriguingly, in these cells, ORP8 was also phosphorylated (Fig. 5A). Serum starvation-stimulated ORP8 phosphorylation was abolished by AMPK inhibitor compound C or KO of AMPK α1/α2 (Fig. 5A and 5B). In addition, co-immunoprecipitation detected the interaction between ORP8 and AMPK, which was promoted by serum starvation and eliminated by compound C (Fig. 5C). Moreover, *in vitro* kinase assay using purified ORP8 and kinase-active AMPK complex confirmed that ORP8 is the direct substrate of AMPK (Fig. 5D).

Phosphorylated ORP8 from *in vitro* kinase assay was analyzed by mass spectrometry. Two conserved residues, Thr54 and Ser65, were suggested as phosphorylation sites (Fig. S5B). Mass spectrometry analysis of ORP8 immunoprecipitated from serum-starved cells and cells treated with AMPK activator AICAR was also performed (Table S2). In untreated cells, Thr54 and Ser65 were included in multiple potential phosphorylation sites, and AICAR and serum starvation increased the stoichiometric phosphorylation of Thr54 (Fig. S5C and S5D). Then, we constructed ORP8 mutants in which Thr54 and/or Ser65 were replaced by alanine via site-directed mutagenesis. In starved transfected cells, the phosphorylation of T54A and T54A/S65A mutants was much lower than that of WT ORP8, and the phosphorylation of S65A mutant was also reduced (Fig. 5E). By creating an antibody that specifically recognizes ORP8 phosphorylated at Thr54, we detected phosphorylation in ORP8-WT precipitates, but not in ORP8-T54A/S65A or ORP8-T54D/S65D precipitates (Fig. 5F). These results confirmed that Thr54 and Ser65 are indeed two phosphorylation sites of AMPK, and Thr54 may be more important.

Next, we determined the effect of phosphorylation on ORP8-mediated lipophagy. We examined whether phosphorylation affects the LD-localization of ORP8. By living cell imaging, we observed the distribution of mCherry-ORP8-T54A/S65A on LDs, which also increased with cell serum starvation (Fig. S5E). Intriguingly, GST pull-down analysis showed that fewer ORP8-T54A, ORP8-S65A or ORP8-T54A/S65A were pulled down from cell lysates by GST-LC3B or GST-GABARAP than ORP8-WT and unrelated ORP8 mutants (Fig. 5G and 5H). Moreover, reintroduction of ORP8-WT but not ORP8-T54A/S65A significantly reduced the content of LDs in serum-starved ORP8-KO cells (Fig. 5I–K), and resulted in the co-localization of LDs and lysosomes (Fig. S5F and S5G). FACS analysis of mCherry-GFP-livedrop also showed that in ORP8-KO cells, compared with ORP8-WT transfection, ORP8-T54A/S65A transfection produced fewer cells with acidified livedrop, indicating reduced LD lysosomal degradation (Fig. 5L). Taken together, these results suggest that AMPK promotes the interaction of ORP8 with LC3/GABARAP by directly phosphorylating ORP8, thereby activating ORP8-mediated lipophagy.

### ORP8 reduces liver lipid deposition in mice

**Figure 6.**
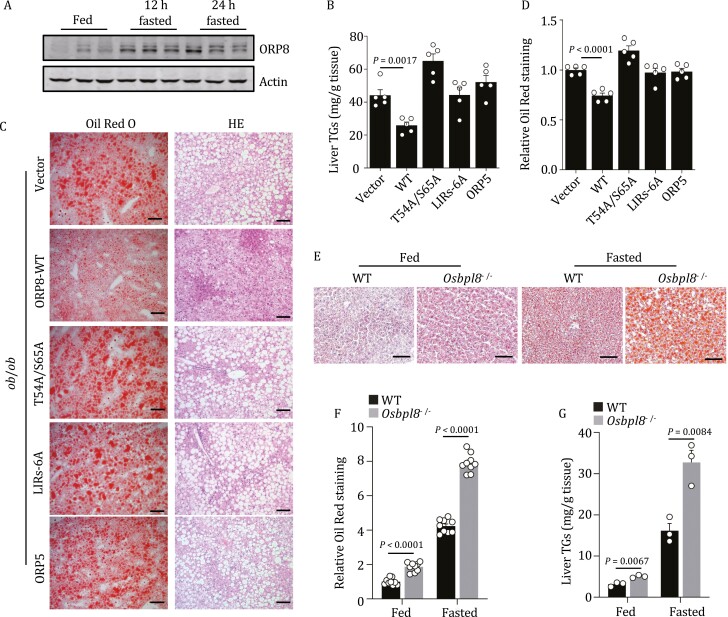
**ORP8 alleviates lipid deposition in mice.** (A) Expression of ORP8 protein in the liver of fasting *ob*/*ob* mice. (B) TG levels in the liver of *ob*/*ob* mice. Mice were intraperitoneally injected with HA-tagged rAAV-vector, rAAV-ORP8-WT, rAAV-ORP8-T54/S65A, rAAV-ORP8-LIRs-6A or rAAV-ORP5. *n* = 5 mice. (C) Oil red O and HE staining of liver tissue of mice treated in (B). Scale bar, 100 μm. (D) Quantification of Oil red O staining area in (C). *n* = 5 views from 5 mice. (E) Oil red O staining of liver tissue of WT and *Osbpl8*^−/−^ mice after 24 h of fasting or no fasting. Scale bar, 100 μm. (F) Quantification of Oil red O staining area in (E). *n* = 9 views from 3 mice. (G) TG levels in the liver of WT and *Osbpl8*^−/−^ mice after 24 h of fasting or no fasting. *n* = 3 mice.

We evaluated the *in vivo* physiological effects of ORP8-mediated lipophagy in *ob/ob* mice. Interestingly, we unexpectedly observed that fasting stimulated the expression of ORP8 protein and mRNA in mouse liver (Figs. 6A, S6A and S6B). We then intraperitoneally injected *ob*/*ob* mice (at 8 weeks) with recombinant adeno-associated virus (rAAV) expressing ORP8-WT, ORP8-T54A/S65A, ORP8-LIRs-6A, or ORP5 (Fig. S6C). Four weeks after injection, only ORP8-WT expression significantly reduced the level of hepatic TG (Fig. 6B) and alleviated the accumulation of LDs in the liver (Fig. 6C and 6D). However, none of these injections significantly reduced plasma TG levels in *ob*/*ob* mice (Fig. S6D), which may reflect more complex regulation of plasma TG.

We also constructed *Osbpl8*^−/−^ mice with CRISPR-Cas9-mediated genome engineering. In these mice, fragment deletion was generated between exon 3 and exon 23 of *Osbpl8* gene. Western blot analysis of mouse liver tissues samples confirmed the loss of ORP8 protein in *Osbpl8*^−/−^ mice (Fig. S6E). In middle-aged *Osbpl8*^−/−^ mice (at 18 weeks) on a normal diet, we detected significant increases in liver LDs (Fig. 6E and 6F) and TG levels (Fig. 6G), although the weight of these mice did not change significantly compared with wild-type mice. After fasting for 24 h, these *Osbpl8*^−/−^ mice showed a significant fatty liver phenotype, with further accumulation of liver LDs (Fig. 6E and 6F) and further elevation of liver TG (Fig. 6G). Taken together, these data suggest that ORP8 plays an important role in LD catabolism *in vivo*.

## Discussion

In this study, through *in vitro* and *in vivo* analysis, we have identified ORP8 as a specific lipophagy receptor. Our results suggest that ORP8 mediates autophagic recognition and degradation of LDs through AMPK-regulated interaction with LC3/GABARAPs. These findings may provide new targets for the intervention and treatment of lipid metabolism related diseases.

Our results showing the localization and recruitment of ORP8 to LDs differ from a recent study showing that ORP8 is undetectable on LDs under long-term OA treatment (Du et al., 2020). This may be due to the inhibition of ORP8 expression and lipophagy by prolonged OA stimulation (Singh et al., 2009; Yang et al., 2010; Jordan et al., 2011). ORP8 shares 58% sequence identity with ORP5. Structurally, both ORP5 and ORP8 contain a lipid interacting ORD domain and a C-terminal TM domain fully embedded in membrane (Olkkonen and Li, 2013; Chung et al., 2015). In contrast to the localization of ORP5-ORD but not ORP5-TM on LDs (Du et al., 2020), we found strong localization of ORP8-TM but not ORP8-ORD on LDs (Fig. 1F and 1G). In addition, the ORP5 mutant with the TM domain of ORP8 can localize to LDs in cells without OA treatment (Fig. S1K). Furthermore, we demonstrated that ORP8 but not ORP5 mediates autophagic degradation of LDs. These observations may indicate different LD targeting mechanisms for ORP5 and ORP8. When ORP5 uses its ORD to contact LDs (Du et al., 2020), ORP8 may translocate to LDs through its C-terminal TM domain. In fact, although the hydrophobic hairpin domain is the most common structural feature of LD-localized integral membrane proteins (Olarte et al., 2020), membrane proteins containing terminal hydrophobic domains not long enough to form hairpin can also localized to LDs (Zehmer et al., 2008; Zhang and Liu, 2019). The molecular mechanism by which ORP8 translocates to LD is currently unclear. We hypothesize that the specific amino acid composition of ORP8 TM domain (more hydrophobic than ORP5 TM domain) may enable it to insert into the phospholipid monolayer. More recently, it was reported that ORP8 can be enriched at mitochondrial associated membrane (MAM)-LD contact sites and contribute to the biogenesis of LDs (Guyard et al., 2022). Possible reason for the different conclusions about the role of ORP8 in LD metabolism may be that we starved cells for a long time to inhibit LD biogenesis and induce lipophagy, instead of removing pre-existing LDs and treating cells with OA for a short time (Guyard et al., 2022), which makes it difficult to detect the degradation of LDs. Our results do not rule out the possibility that ORP8 plays a role in LD biogenesis, especially in OA-treated cells. However, we speculate that this effect of ORP8 is likely not dominant in LD metabolism, because although ORP8-KD reduces the biogenesis of LD, LDs can still be produced in the cells, and the number gradually approaches that of wild-type cells over time (Guyard et al., 2022). By comparison, our data from ORP8-KO cells combined with *in vivo* results using ORP8-KO mice strongly suggested that deletion of ORP8 actually leads to LD accumulation.

The role of AMPK in autophagy initiation has been intensively studied. Recently, it was reported that AMPK also participates in lipophagy (Jordan and Randall, 2017; Zhang et al., 2020). However, the function of AMPK in these studies is still limited to the induction of phagophore formation. We determined that ORP8 is the phosphorylation substrate of AMPK, which confirmed the participation of AMPK and revealed the mechanism of AMPK in lipophagy regulation. It is possible that the function of AMPK in ORP8-mediated LD encapsulation is coordinated with its role in phagophore formation, thereby ensuring LD degradation through lipophagy when energy is insufficient. Nevertheless, our findings support a function of AMPK in lipophagy by facilitating the recognition and encapsulation of LDs, which is consistent with a recent report showing that AMPK contributes to mitophagy by inducing ULK1-mediated Parkin activation (Hung et al., 2021). Considering that AMPK can be activated by different cues and starvation strongly triggers selective autophagy of different organelles, similar effects and mechanisms of AMPK may be involved in autophagic clearance of other intracellular structures.

It is worth noting that the Thr54 and Ser65 in ORP8 are not in a classical AMPK phosphorylation consensus motif. Instead, Ser97 residue is located in a perfect AMPK consensus site. However, our mass spectrometry analysis of ORP8 from cells and from *in vitro* kinase assay never showed a potential phosphorylation at Ser97. In addition, the created ORP8-S97A mutant did not show reduced phosphorylation as did the ORP8-T54A/S65A (data not shown). Moreover, we tested whether ORP8 can be phosphorylated by other nutritional stress-sensitive kinases that may be regulated by AMPK, including p38, JNK, and GSK3. Using specific inhibitors of these kinases, we found that none of them reduced ORP8 phosphorylation or ORP8 phosphorylation at Thr54 (data not shown). This suggests that Thr54 and Ser65 in ORP8 are non-canonically phosphorylated by AMPK, as has been found in other cases (Vernia et al., 2009; Li et al., 2015; Naito et al., 2020).

As a resident protein of ER, the interaction between ORP8 and LC3/GABARAPs suggests that ORP8 may play a potential role in ER-phagy. However, our results showed that the deletion of ORP8 did not affect the content of calnexin (Fig. 2E). It is possible that because ORP8 contains only one non-penetrating TM, it cannot disrupt the ER membrane by oligomerization as is known for ER-phagy receptors with multiple transmembrane domains, such as FAM134B (Khaminets et al., 2015).

## Materials and methods

### Antibodies and reagents

Antibodies: Anti-ORP8, anti-ORP5, anti-PLIN2 and anti-goat IRDye 800CW secondary antibodies were obtained from Abcam; anti-OSBP from Invitrogen; anti-GAPDH from Santa Cruz Biotechnology; anti-LC3B for Western blot, anti-actin and anti-phosphoserine/threonine from Sigma-Aldrich; anti-AMPK and anti-phospho-AMPK (Thr172) from Cell Signaling Technology; anti-ORP2 and anti-p62 from Proteintech; anti-ATGL from ABclonal Technology; anti-HA, anti-Myc and anti-flag from Medical and Biological Laboratories, anti-LC3B for immunostaining from Cosmo Bio; anti-phospho-ORP8 (Thr54) was custom-made by HUABIO; anti-mouse IRDye680RD and anti-rabbit IRDye800CW secondary antibodies for Western blot were from LI-COR Biosciences; and anti-mouse Alexa Fluor 488-tagged and anti-rabbit Alexa Fluor 546-tagged secondary antibodies for immunostaining were from Molecular Probes.

Reagents: Glutathione Sepharose 4B beads were obtained from GE Healthcare; protein G agarose and protein A agarose from Santa Cruz Biotechnology; GFP-TRAP magnetic beads from Chromotex; and Anti-HA affinity beads from Biotool. Triacsin C was obtained from Abcam; BODIPY 493/503, BODIPY Red C12, Lipofectamine 2000, Lipofectamine 3000 and Trizol from Invitrogen; Lipi-blue from Dojindo; Fluoromount-G from Southern Biotech; and QuikChange II XL from Agilent. The 1st strand cDNA synthesis kit was from TOYOBO. The HiScript II Q Select RT SuperMix was obtained from Vazyme. Lipidtox was gratefully received as a gift from Dante Neculai. All other reagents were from Sigma-Aldrich unless indicated otherwise.

### Plasmid constructs

For expression in *Escherichia coli*, cDNA encoding LC3A, LC3B, GABARAP, GABARAPL1, GABARAPL2 were cloned into pGEX-5X-1; cDNA encoding ORP8 was cloned into modified pGEX-4T-1 with a TEV restriction site.

For expression in mammalian cells, ORP8 and ORP5 cDNA were cloned into pXF4H (with 2× HA), pEGFP-C1 and pmCherry-C1. APEX2-ORP8 was constructed by inserting APEX2 cDNA into HA-ORP8. GFP-2A-ORP8 was constructed by inserting T2A cDNA into GFP-ORP8. MCherry-GFP-livedrop were modified from mCherry-livedrop. PEP-ORP8-KO and pEP-ATGL-KO were constructed by inserting the gDNA sequence of human ORP8 (GCCCAAATAGATTGCTCCAA) and ATGL (CATTCTCGCCGT CTGACACG) into a pEP-KO Z1779 vector. PEP-ORP8-KI was constructed by inserting the gDNA sequence of human ORP8 (ACAGAATGGCTGCACATTAA) targeting the knock in site into a pEP-330x plasmid. The recombinant construct for creating Cherry ORP8 knock in HeLa cells was generated by taking a 1 kb fragment before or after the sgRNA and inserting Cherry in the N-terminal ATG site of human ORP8 genomic DNA in psx1184 plasmid. QuikChange II XL was used to generate the series site-mutants on the basis of HA-ORP8, mCherry-ORP8 and GFP-2A-ORP8, according to the manufacturer’s instructions. GFP-ORP5, GFP-OSBP and ORP2-GFP were gifts from Vesa M. Olkkonen.

### Cell culture, construction, transfection and treatment

HeLa, HEK293, HEK293T, Huh7 and MEF cells were cultured in Dulbecco Modified Eagle Medium (DMEM) (Gibco) containing 10% fetal bovine serum (Gibco) in a 37°C incubator with 5% CO_2_. Plasmid transfection was performed with Lipofectamine 2000 or Lipofectamine 3000 according to the manufacturer’s instructions.

ORP8 KO HeLa and HEK293 cells, as well as ATGL KO and ORP8/ATGL DKO HeLa cells, were generated by transfecting pEP-ORP8-KO or pEP-ATGL-KO plasmids, respectively, then via selection with puromycin. Monoclonal cell lines were established by limited dilution method. p62/NDP52/NBR1/OPTN/TAX1BP1 KO HeLa cell line was a gift from Hanming Shen. AMPK α1/α2 DKO MEF cell line was a gift from Zongping Xia.

For generating Cherry-ORP8 knock in cells, HeLa cells were co-transfected with pEP-ORP8-KI plasmid containing the gDNA and psx1184 plasmid containing homology arms and mCherry sequence. After puromycin selection, positive clones were screened by flow cytometry and by direct observation using confocal microscopy.

HeLa and HeLa ORP8 KO cell lines stably expressing mCherry-GFP-livedrop, HeLa cell line stably expressing Flag-LC3, as well as the HEK293 cell line stably expressing GFP-LC3 were generated by transient transfection and selection with G418.

For the RNAi experiments, siRNA duplexes were transfected for 48 h. The following siRNA duplexes (GenePharma) were used: negative control siRNA: UUCUUCGAACGUGUCACGUTT; ORP8 siRNA: GGAGCUUGGUGGAACAGUC AAUAUU; ORP5 siRNA: CCCTGCCCAGCAGCTACCTGATCTT; ATG7 siRNA: CAGUGGAUCUAAAUCUCAAACUGAU.

For serum starvation, cells were washed twice with PBS and transferred to DMEM without serum for 24 h unless otherwise indicated. Other chemical treatments were described in Figure legend in detail.

### Mice

Male WT and *ob*/*ob* C57BL/6 mice (8 weeks of age) were purchased from GemPharmatech Co., Ltd (Nanjing, Jiangsu, China). Male WT and *Osbpl8*^−/−^ mice (18-weeks of age) were constructed by GemPharmatech Co., Ltd (Nanjing, Jiangsu, China) based on CRISPR/Cas9 technique. All mice were housed under a 12 h light/dark cycle with food and water ad libitum, unless otherwise indicated. For fasting, mice were starved without food, but with free access to water, for 12 h or 24 h. All animal experiments were approved by the Animal Care and Use Committee of the animal center at Zhejiang University.

### Confocal microscopy and image analysis

For imaging of fixed cells expressing fluorescent proteins, cells grown on coverslips were fixed with 2% formaldehyde or 4% paraformaldehyde (PFA) for 15 min at room temperature and washed three times with PBS. Fixed cells were stained with or without lipid dyes according to the experimental design and mounted with Fluoromount-G. For LDs staining, cells were incubated with PBS containing 1 μg/mL of Bodipy 493/503 or LipidTox or Lipi-blue for 1 h. For immunostaining in Fig. 4H, fixed cells were permeabilized with 0.1% Triton X-100 in PBS after fixation and PBS washing, and then blocked with 5% FBS, followed by incubated with primary antibody and secondary antibody. Cells were then mounted with Fluoromount-G.

Confocal images were captured in Airyscan high-resolution mode on a LSM 880 confocal microscope system equipped with a 63× Plan Apochromat 1.4 NA objective (Carl Zeiss). For z-stack imaging, images with 0.2 μm per section were obtained. For live-cell imaging, cells were grown on live-cell chambers (Lab-Tek) and observed with a LSM 880 microscope at 37°C with 5% CO_2_ atmosphere.

Images were processed in ImageJ Fiji. The number and total area of LDs were measured through ImageJ Fiji. 3D surface reconstitution of LDs was performed in Imaris 9.3.1. Statistics of Z-stack images were also performed in Imaris 9.3.1. LDs stained with fluorescent lipid dyes were detected in surface mode with background subtraction method, and the numbers and volumes of LDs were automatically measured. For ease of display, Z-stack images were processed with maximum intensity projection in Imaris.

### LD purification

LD purification was performed according to the previously reported protocol (Ding et al., 2013). Briefly, cells incubated with 200 μmol/L OA overnight to induce LDs which were then homogenized with a Dounce Tissue Grinders and resuspended in Buffer A (20 mmol/L tricine, 250 mmol/L sucrose and 1 mmol/L EDTA, pH 7.8). They were then centrifuged at 4°C with 3,000 ×*g* for 10 min. Post-nuclear supernatant was then transferred to a SW41 ultracentrifugation tube. Buffer B (20 mmol/L HEPES, 100 mmol/L KCl, 2 mmol/L MgCl_2_, pH 7.4) was loaded on the top of the post-nuclear supernatant. Ultracentrifugation with 182,000 ×*g* at 4°C was performed for 1 h so that the LD fraction would be at the top of the tube. To extract LD protein, acetone was added into the purified LDs followed by centrifugation to precipitate the LD protein. The sediment was dissolved in SDS sample buffer and analyzed using Western blot.

### Western blot and immunoprecipitation

Briefly, cells were lysed using laemmeli buffer (60 mmol/L Tris-HCl, 10% glycerol, 2% SDS and 1 mmol/L EDTA, pH 6.8) supplemented with complete protease inhibitor cocktail (Roche) and phosphatase inhibitors (Sangon). Proteins were prepared in 4× SDS sample buffer (240 mmol/L Tris-HCl, 40% glycerol, 8% SDS, 4 mmol/L EDTA and 0.04% bromophenol blue, pH 6.8) and run on 6%–12% SDS-polyacrylamide gels. After electrophoresis, the samples were transferred to a PVDF membrane (Millipore). The membrane was blocked with 5% BSA in TBST and incubated with the primary antibody at 4°C overnight and the secondary antibody at room temperature for 1 h. Finally, the membrane was scan and analyzed using the Odyssey infrared imaging system (LICOR Biosciences). Image J was used for Quantification of the Western blot bands.

For immunoprecipitation, cells were lysed in NP-40 buffer (50 mmol/L HEPES, 150 mmol/L NaCl, 10% glycerol, 1% NP-40, 1 mmol/L EDTA, 1 mmol/L EGTA, pH 7.4) supplemented with complete protease inhibitor cocktail and phosphatase inhibitors. The appropriate antibody was added to the lysates and incubated overnight at 4°C. Then Protein A or Protein G Sepharose beads were added into the mixture to precipitate the antigen-antibody complexes. After washing with lysis buffer, the immunocomplexes were analyzed using Western blot.

### Triglyceride measurement

Triglyceride was measured using a GPO-PAP triglyceride kit (NJJCBIO) according to the manufacturer’s instructions. The TG level in the cells was standardized by the protein level of the same sample as measured by a BCA kit (Sangon) and the results were presented as μg/mg protein. For the measurement of mouse liver triglycerides, the liver tissues were weighed and homogenized in 9 volumes of chloroform: methanol mixture (2:1, *v*/*v*). The homogenate was rotated at RT for 12 h, then vortexed and centrifuged at 2,500 ×*g* for 10 min. The lower phase containing lipids was collected and dried at 55°C. The dried pellet was resuspended in 2 mL isopropanol and measured using the GPO-PAP triglyceride kit. Results are presented as mg/g tissue.

### Protein expression and purification

GST-ORP8 was expressed in *Escherichia coli* BL21 at 16°C overnight. Cells were collected and lysed by sonication in a NP-40 buffer supplemented with protease inhibitor, followed by centrifugation at 12,000 ×*g* for 15 min. Glutathione Sepharose 4B beads pre-equilibrated with NP-40 buffer were added to the supernatant and rotated at 4°C overnight. The beads were washed 4 times with NP-40 buffer and once with a TEV cleavage buffer (10 mmol/L Tris-HCl, 150 mmol/L NaCl, 1 mmol/L DTT, pH 8.0). TEV protease was added in a TEV cleavage buffer and incubated at 4°C overnight to cleave off ORP8 from bead-bound GST. TEV was depleted using nickel beads (Sigma). Purified ORP8 was further dialyzed with PBS or other buffer for subsequent experiments and analyzed using Coomassie blue staining. GST-ATG8 family proteins were expressed in *Escherichia coli* BL21 at 37°C for 3 h and purified similarly without release from beads.

### GST pull-down assay

Purified GST-ATG8 family proteins were added into cell lysates or into purified ORP8 protein in NP-40 buffer, and incubated at 4°C for 2 h. Centrifugation was conducted at 1000 ×*g* for 2 min to precipitate the bead-protein complexes. The complexes were then washed with NP-40 buffer and PBS, and finally boiled in SDS sample buffer and analyzed using Western blot.

### Electronic microscopy and APEX EM

For common transmission electronic microscopy, cells were fixed in 2.5% glutaraldehyde at 4°C overnight, followed by post-fixation with 1% OsO_4_ for 1 h. Cells were stained by 2% uranyl acetate for 30 min and processed with a dehydration series using 50%–100% ethanol and incubation in 100% acetone. Samples were then infiltrated with 1:1 acetone and epoxy resin for 2 h and 1:3 acetone and epoxy resin overnight, followed by polymerization in resin at 60°C for 2 days. Ultrathin sections were then prepared using a Leica UC7 ultramicrotome. The sections of the samples were observed under a Tecnai G2 Spirit transmission electron microscope at 120 kV.

APEX EM was performed as previously described (Du et al., 2020). Briefly, after fixation in 2.5% glutaraldehyde at 4°C overnight, cells were washed with PBS and 1 mg/mL DAB in PBS. They were then incubated with 1 mg/mL DAB with 0.02% (*v*/*v*) H_2_O_2_ for 30 min, followed by post-fixation with 1% OsO_4_ for 30 min. Samples were directly processed with gradient dehydration in 50%, 70%, 90% and 100% ethanol and infiltrated with 1:1 ethanol and epoxy resin and 1:3 ethanol and epoxy resin for 2 h. Finally, samples were embedded in epoxy resin. The following steps are similar to common transmission electronic microscopy described above.

For APEX EM assay of purified LDs, LDs were purified from cells expressed HA-APEX2-ORP8 according to the method described above. Then, the purified LDs were mixed with 12% gelatin (0.1 mol/L PBS, pH 7.2) in a 1:1 ratio at 40°C. LDs-gelatin mixture was rapidly cooled to solidify. The sample was cut into ~1 mm^3^ blocks and fixed in 2.5% glutaraldehyde at 4°C overnight. The following steps are similar to APEX EM described above.

### Fluorescent FA pulse-chase assay

The fluorescent FA pulse-chase assay was performed according to previously described protocol with minor modification (Rambold et al., 2015). Briefly, HeLa cells transfected with GFP-LC3 were treated with 1 μmol/L Bodipy Red C12 in DMEM containing 10% FBS for 16 h. Cells were triple washed with PBS and incubated in DMEM containing 10% FBS for 1 h and directly imaged using a LSM 880 microscope at 37°C in a 5% CO_2_ atmosphere. The cells were serum-starved for 24 h and again subjected to live-cell imaging.

### *In vitro* binding assay of LDs and LC3-membranes

Purification of GFP-LC3-positive membranes was performed as previously described (Gao et al., 2010). Briefly, HEK293 cells stably expressing GFP-LC3 were starved in EBSS, then collected in Buffer I (250 mmol/L sucrose, 1 mmol/L EDTA, 20 mmol/L HEPES, pH 7.4) supplemented with protease inhibitor. Cells were disrupted by passing 15 times through a 22-gauge needle. The lysate was centrifuged at 800 ×*g* for 10 min. Post-nuclear supernatant was further centrifuged at 10,000 ×*g* for 20 min. After washing twice in PBS, the pellet was suspended in Buffer II (2 mmol/L EDTA, 3% BSA in PBS) and incubated with GFP-TRAP magnetic beads overnight. GFP-LC3 membranes immortalized on GFP-TRAP magnetic beads were then incubated overnight at 4°C with equivalent purified Lipi-blue-stained LDs which has been quantitated using TG concentration. After incubation, the beads were triple washed with PBS and suspended in 200 μL PBS. They were then mixed with mounting buffer and loaded on a slice for confocal microscopy.

### *In vitro* kinase assay

Purified ORP8 protein (0.5 μg) in kinase assay buffer (60 mmol/L Tris, 10 mmol/L MgCl_2_, 0.1 mmol/L Na_3_VO_4_, 5 mmol/L β-glycerophosphate, 5 mmol/L DTT and 100 μmol/L AMP) was incubated with kinase-active AMPK complex (0.2 μg) (SignalChem) at 30°C in the presence or absence of 100 μmol/L ATP or 50 μmol/L compound C for 1 h. SDS sample buffer was then added to terminate the reaction. The samples were analyzed using Western blot with a phosphoserine/threonine antibody.

### HPLC–MS/MS

To screen LC3 interaction proteins or phosphorylation sites of HA-ORP8 immunoprecipitated from cells, GST-LC3 bound glutathione sepharose 4B beads or HA-ORP8 bound agarose beads were precipitated. On-bead digestion was performed with MS-grade trypsin (Promega) in 50 mmol/L ammonium bicarbonate at 37°C overnight. For the identification of the *in vitro* phosphorylation sites in ORP8, the phosphorylated ORP8 from the *in vitro* kinase assay was separated using SDS-polyacrylamide gel electrophoresis and stained with Coomassie blue. The band of ORP8 was cut down and performed in-gel digestion with trypsin at 37°C overnight.

After desalting, digested peptides were loaded on a capillary reversed-phase C18 column (15 cm in length, 100 μm ID × 360 μm OD, 3 μm particle size, 100 Å pore diameter) which was connected to an Agilent HPLC1260 system. Analysis was performed at a flow velocity of 300 nL/min with a 180 min HPLC gradient from 0 to 100% of 0.1% formic acid in an acetonitrile buffer. After ionization, the eluted peptides were introduced into a Q-Exactive mass spectrometer (Thermo Fisher Scientific) via a nano-spray source. An Orbitrap analyzer was used to perform a full-scan MS spectra survey with a resolution of *r* = 70,000 at *m/z* 400.

The acquired MS/MS data were analyzed against a UniProtKB human or *E*. *coli* containing target proteins using Proteome Discoverer 2.4 or PEAKS studio X. Precursor mass tolerance was set to ± 20 ppm and fragment mass tolerance was set to ± 0.2 Da. Carbamidomethylation of cysteine (+57.021 Da), oxidation of methionine (+15.995 Da) and phosphorylation of Serine\threonine\tyrosine was set as variable modifications. All identified proteins had an FDR of ≤1%, which was calculated at the peptide level.

### Relative quantification of phosphorylation sites in mass spectrometry analysis

Relative Quantification of phosphorylation sites of HA-ORP8 immunoprecipitated from cells treated with AICAR or serum starvation, was performed as previously described with minor modification (Egan et al., 2011). Briefly, for each phosphorylation site, a ratio of total ion counts (TIC) signal of phosphorylated peptide to the total peptide was calculated as $R_{PO_{4}}$. Then the $R_{PO_{4}}$ of AICAR or serum starvation sample were compared to the same phosphopeptide $R_{PO_{4}}$ of control sample.

### Mouse experiments

Recombinant adeno-associated virus (rAAV) packing HA-tagged ORP8 WT, ORP8 T54A/S65A, ORP8 LIRs mutant and ORP5 vectors were produced in HEK293T cells (Vigenebio). Eight weeks old *ob*/*ob* mice were intraperitoneally injected with the 10^12^ vg these viruses or a control AAV9 virus, respectively. Four weeks after viral infection, fresh blood and liver tissues were isolated from mice after anesthesia using Avertin. For oil red O staining and immunostaining, liver tissues were immediately frozen in liquid nitrogen. Samples were then embedded in Tissue-Tek OCT and sectioned on a CryoStar NX50 (Thermo). For HE staining, fresh liver tissues were fixed in 4% formaldehyde (Sangon) for 48 h. Samples were then embedded in paraffin and sectioned on a paraffin slicing machine (LEICA RM2235).

### RNA isolation and quantitative PCR

Trizol was used to isolate total RNA from liver tissue. RNA reverse transcription was performed using a 1st strand cDNA synthesis kit. qPCR analysis was performed using HiScript II Q Select RT SuperMix on a real-time PCR machine (Bio-Rad). ORP8 qPCR primer sequence: ATGGAGGCAGCCTTAGCAGA; GAPDH qPCR primer sequence: AGGTCGGTGTGAACGGATTTG. The Comparative CT method was used to quantify each mRNA.

### Oil Red O and HE staining

For Oil Red O staining, frozen liver section was washed with PBS and stained with Oil Red O solution for 15 min. After differentiation with 60% ethanol, the sample was rinsed with water and mounted. For HE staining, fixed liver section was deparaffinized in xylene and rehydrated using graded concentrations of ethanol. After staining in hematoxylin then eosin solution, the sample was mounted on a slide and imaged using an Olympus bx61 microscope.

### Flow cytometry analysis of lipophagy flux

Flow cytometry of lipophagy flux was performed using a CytoFLEX LX Flow Cytometer (Beckman) and subsequent analysis was performed using the FlowJo software. To prevent GFP quenching by cell fixation, experiments were performed using live cells. The intensities of GFP and Cherry signal of WT cells expressing mcherry-GFP-livedrop treated with CQ for 12 h at fed conditions were used as a reference to define the gate for zero lipophagy. The measurement of lipophagy flux was based on the shift of cell population into the lipophagy positive gate (mCherry signal > GFP signal). An average of 10,000–50,000 cells were analyzed under each condition.

### Statistical analysis

All data are presented as mean ± SEM. The exact *P* values were all for two-sided tests and provided in the figures. All data presented are based on at least three independent experiments. Data were tested for normality. Unpaired Student’s *t*-tests were performed on the data conforming to the normal distribution. If the data do not conform to the normal distribution, Mann–Whitney *U* tests were performed. Graphpad prism 9 software was used for all statistical analysis.

## Supplementary Material

pwac063_suppl_Supplementary_Materials

pwac063_suppl_Supplementary_Table_S1

pwac063_suppl_Supplementary_Table_S2

## Data Availability

MS data have been deposited in PRIDE. The accession code of MS data related to Extend Data Fig.1A is PXD036200. The accession code of MS data related to Extend Data Fig. 5B is PXD036201. The accession code of MS data related to Extend Data Fig. 5C and 5D is PXD036204. Any other data are available from the corresponding authors upon request.

## Abbreviations

AAV: adeno-associated virus
AMPK: adenosine 5ʹ-monophosphate activated protein kinase
APEX: ascorbate peroxidase
ATG7: autophagy related gene 7
ATGL: adipose triglyceride lipase
Baf A1: bafilomycin A1
CC: compound C
CQ: chloroquine
EBSS: Earle’s balanced salt solution
ER: endoplasmic reticulum
FACS: fluorescence-activated cell sorting
FAM134B: family with sequence similarity 134 member B
GABARAP: GABA type A receptor-associated protein
GSK3: guanosine kinase 3
JNK: c-Jun NH2-terminal kinase
KD: Knockdown
KO: knockout
LAMP1: lysosomal associated membrane protein 1
LC3: microtubule associated protein 1 light chain 3
LDs: lipid droplets
LIR: LC3 interaction regions
NBR1: ubiquitin-associated (UBA)/TS-N domain-containing protein/ octicosapeptide/Phox/Bemp1 (PB1) domain-containing protein
NDP52: nuclear domain 10 protein 52
OA: oleic acid
OPTN: optineurin
ORD: OSBP-related domain
ORP8/Osbpl8: oxysterol-binding protein related protein 8
OSBP: oxysterol-binding protein
PI4P: phosphatidylinositol 4-phosphate
PH: pleckstrin homology
PLIN2: perilipin2
PS: phosphoserine
SQSTM1: Sequestosome-1
SS: serum starvation
TAX1BP1: Tax1-binding protein 1
TG: triglycerides
TM: transmembrane
TrC: triacsin C

## Declarations

The authors declare no competing interests related to the content of this article. All animal experiments were approved by the Animal Care and Use Committee of the animal center at Zhejiang University. All experiments conformed to ethical regulations. All authors participated in this study and gave their consent to publish it. Any data are available from the corresponding authors upon request. No new code was generated in this study. All used softwares are publicly available.
