# Supplementary material for: ORP8 acts as a lipophagy receptor to mediate lipid droplet turnover
Source: Protein Cell. 2022 Dec 7;14(9):653–67. doi: 10.1093/procel/pwac063 (PMC10501187; doi:10.1093/procel/pwac063)
Supplement: pwac063_suppl_Supplementary_Materials [file pwac063_suppl_supplementary_materials.pdf]

Supplementary Data

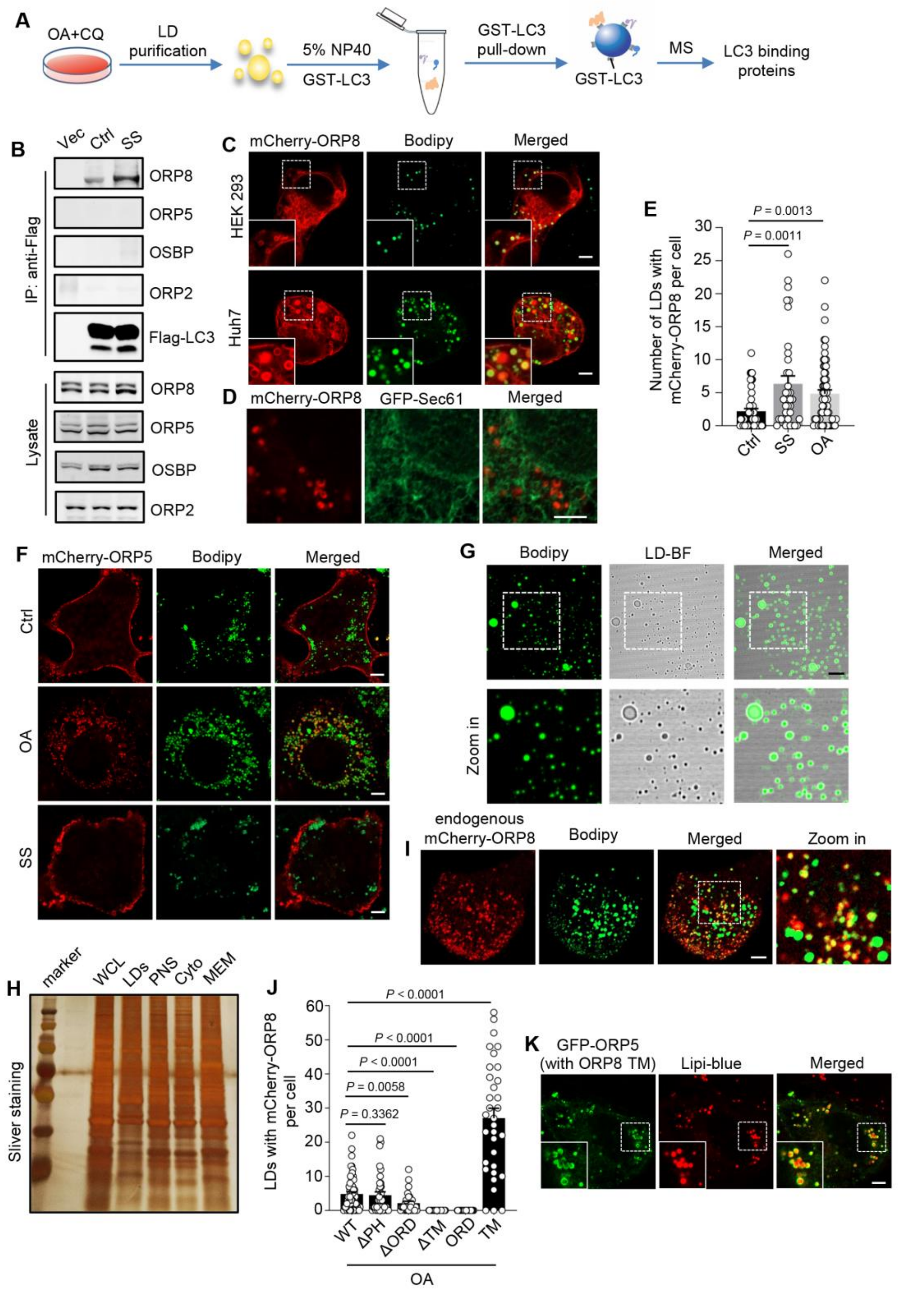

Figure S1. The LD distribution of ORP8.

(A) Scheme depicting the workflow for identifying LC3-binding proteins on LDs. LDs

were purified from HeLa cells treated with 200 μM OA and 10 μM CQ overnight and lysed with 5% NP40 lysis buffer. The diluted lysates (containing 0.5% NP40) were then incubated with purified GST or GST-LC3B immobilized on GSH beads. Proteins associated with the beads were analyzed by tandem mass spectrometry. Proteins only identified in the GST-LC3B group or proteins with ≥ 5-fold enrichment over the GST group were counted as positive and listed in (Table S1). (B) Co-precipitation of endogenous OSBP family proteins with Flag-LC3 from Flag-LC3-stable HeLa cells with or without serum starvation for 24 h. (C) Live-cell images of HEK 293 and Huh7 cells transiently expressing mCherry-ORP8. Cells were treated with 200 μM OA for 6 h and stained with Bodipy. (D) Confocal images of HeLa cells expressing mCherry-ORP8 and GFP-Sec61. (E) Statistics of the number of LDs with mCherry-ORP8 localization per cell in (Fig. 1A). n = 30 cells. (F) Live-cell images of HeLa cells transiently expressing mCherry-ORP5. The cells were OA-treated or serum-starved for 6 h and stained with Bodipy. (G) Purified LDs under fluorescence and bright field microscope, related to (Fig.1B). (H) Silver staining on SDS-PAGE to show the protein loading of cell fraction samples in (Fig. 1B). (I) Endogenous ORP8 was labeled with mCherry using CRISPR-Cas9 mediating genome engineering. Representative live-cell image of the genetically modified cells was shown. The cells were OA-treated for 6 h and stained with Bodipy. (J) Quantification of the number of LDs with mCherry-ORP8 localization per cell in (Fig. 1F and G). n = 30 cells. (K) Representative confocal images of Hela cells transiently expressing GFP-ORP5 (with ORP8 TM domain). Cells were stained with Lipi-blue. OA, oleate acid; SS, serum starvation. BF, bright field. Scale bar, 5 μm.

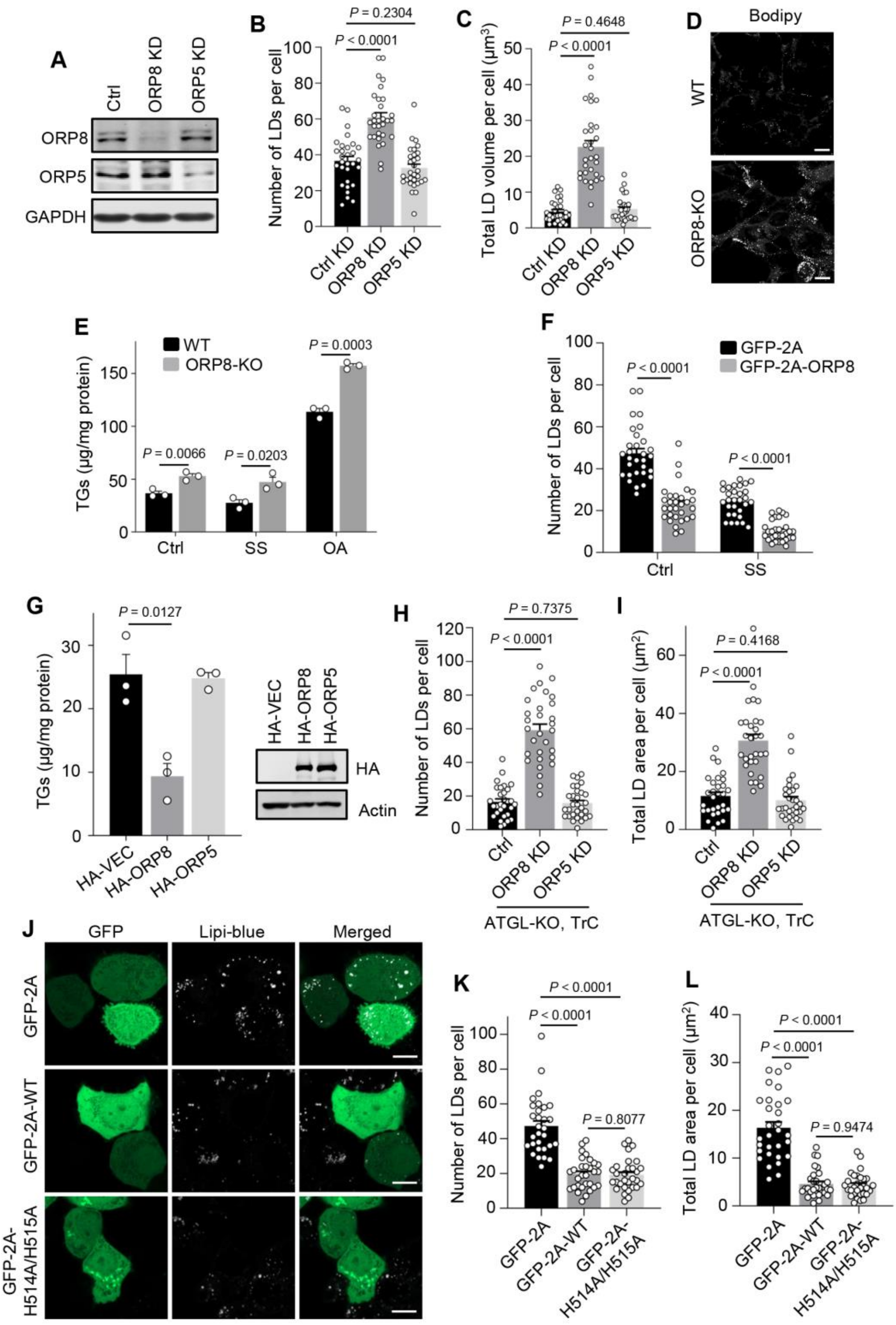

Figure S2. ORP8 promotes LD degradation independently of lipolysis.

(A) Knockdown efficiency of ORP8 and ORP5, related to (Fig. 2A). (B and C) Quantification of the number (B) and total volume (C) of LDs in (Fig. 2A). n = 30 cells. (D) Confocal images of Bodipy-stained WT or ORP8-KO HEK293 cells treated with

serum starvation for 24 h. Scale bars, 10 μm. (E) TG levels in WT or ORP8-KO HEK293 cells treated with or without serum starvation or 200 μM OA for 24 h. The data is quantification of three independent experiments. (F) Quantification of the number of LDs per cell in (Fig. 2D). n = 30 cells. (G) Left: TG levels in negative control HeLa cells or HeLa cells overexpressing HA-ORP8 or HA-ORP5. The data is the quantification of three repeated samples. Right: The expression level of HA-ORP8 or HA-ORP5 was analyzed by Western blot. (H and I) Quantification of the number (H) and total area (I) of LDs per cell in (Fig. 2H). n = 30 cells. (J) Confocal images of HeLa cells transfected with GFP-2A, GFP-2A-ORP8 WT or GFP-2A-ORP8-H514A/H515A. Cells were stained by Lipi-blue. Scale bar, 10 μm. (K and L) Quantification of the number (K) or total area (L) of LDs per cell in (J). n = 30 cells. SS, serum starvation; OA, oleate acid; TrC, triacsin C.

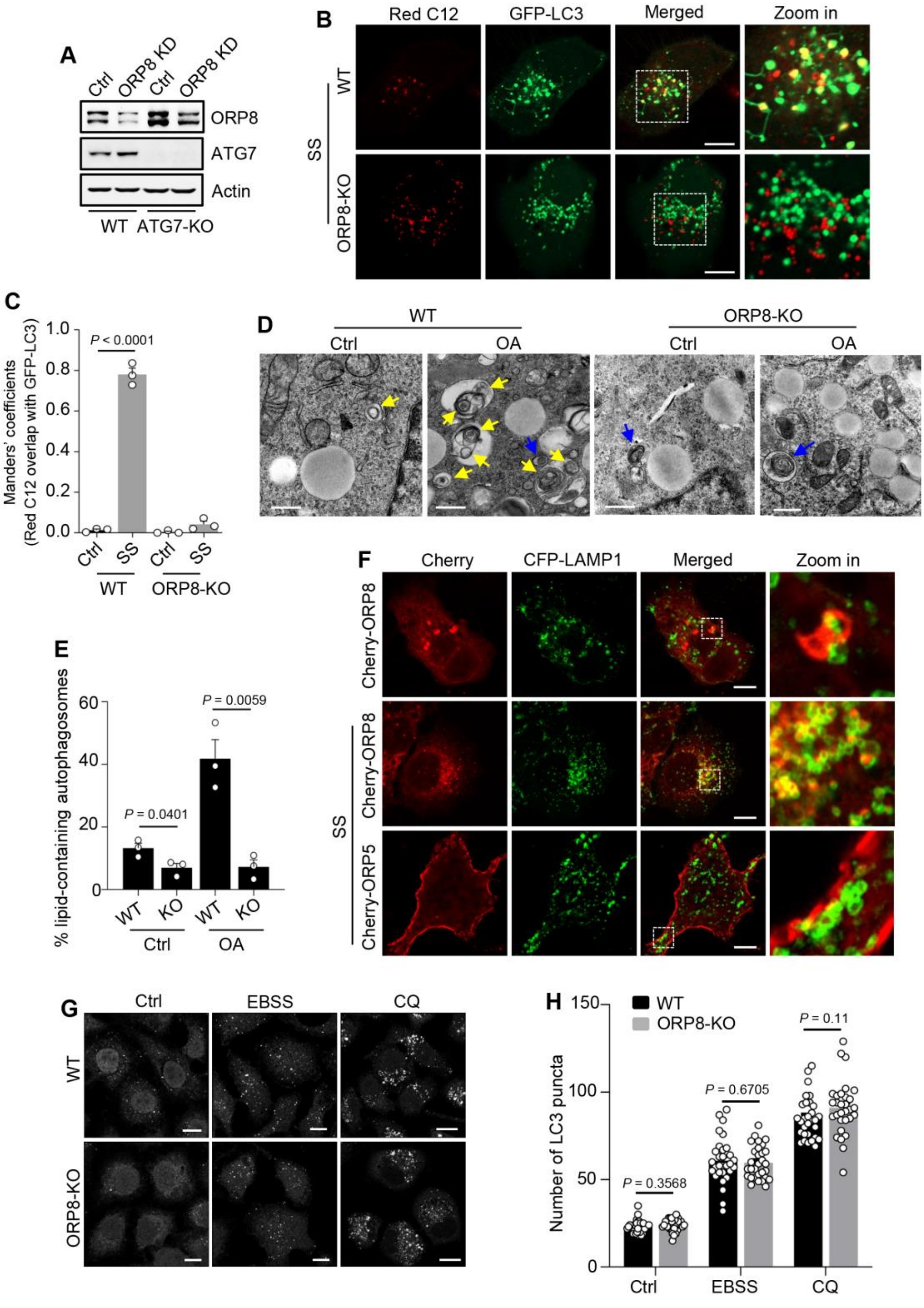

Figure S3. OPR8 regulates lipophagy without affecting non-selective autophagy. (A) Expression of ORP8 and ATG7 in WT or ATG7 KO MEFs treated with or without ORP8 siRNA, related to (Fig. 3A). (B) Live-cell images of GFP-LC3-expressing HeLa cells pulsed with 1 μM Red C12 overnight and serum-starved for 24 h. (C) Co-

localization analysis of Red C12 and GFP-LC3 in (B). Manders' coefficients were used to show the co-localization level. (D) TEM images of WT or ORP8-KO HeLa cells with or without 200 μM OA treatment for 6 h. The yellow arrow indicates lipid-containing autophagosomes, and the blue arrow indicates autophagosome/autolysosome. (E) Quantification of the ratio of lipid-containing autophagosomes to total autophagosomes in (D). n > 130 autophagosomes in three independent experiments. (F) Confocal images of HeLa cells expressing CFP-LAMP1 transfected with mCherry-ORP8 or mCherry-ORP5. Cells were treated with or without serum starvation for 24 h. (G) Confocal images of endogenous LC3 in WT or ORP8-KO HeLa cells treated with or without EBSS or 50 μM CQ for 6 h. (H) Quantification of the number of LC3 puncta in (G). n = 30 cells. SS, serum starvation; OA, oleate acid; EBSS, Earle's balanced salt solution, CQ, chloroquine. Scale bar, 10 μm (B, F, G), 0.5 μm (D).

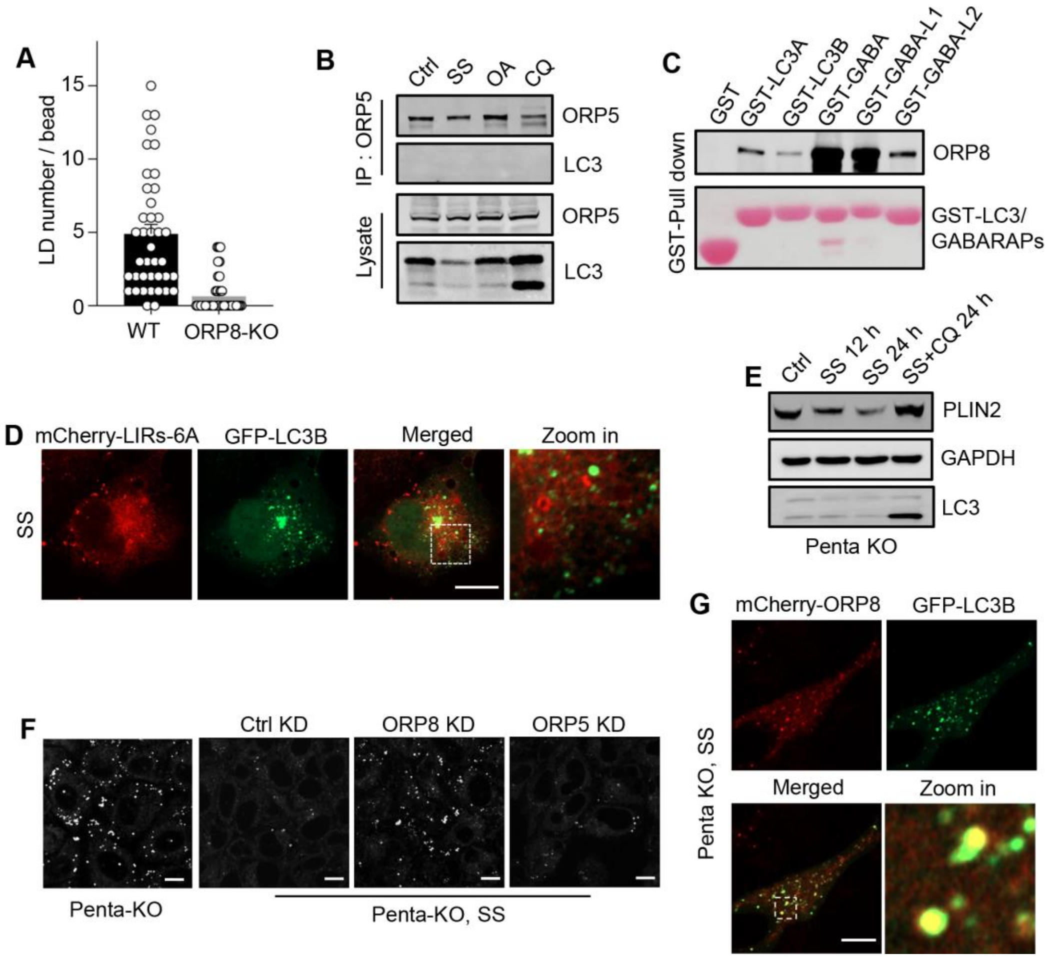

Figure S4. OPR8 mediates lipophagy independently of Ub-binding autophagy adaptors.

(A) Quantification of the LD number per bead in (Fig 4B). n = 40 beads. (B) Endogenous LC3 could not be co-immunoprecipitated with ORP5 in HeLa cells. The cells were serum-starved for 24 h, or treated with 200 μM OA or 50 μM CQ for 6 h. (C) Each indicated purified ATG8 family protein was incubated with purified ORP8, then they were pulled down and the bound ORP8 was analyzed by Western blot. (D) Confocal images of HeLa cells expressing mCherry-ORP8 LIRs-6A and GFP-LC3. Cells were treated with serum starvation for 24 h. (E) Expression of PLIN2 in p62/NDP52/NBR1/OPTN/TAX1BP1-KO (Penta-KO) cells treated with serum starvation or 50 μM CQ for the indicated time. (F) Confocal images of Penta-KO cells with or without KD of ORP5 or ORP8. Cells were serum-starved for 24 h and stained

with Bodipy. (G) Confocal images of Penta-KO cells expressing mCherry-ORP8 and GFP-LC3. Cells were serum-starved for 24 h and stained with Bodipy. LIR, LC3-interacting region; SS, serum starvation. Scale bar, 10 μm.

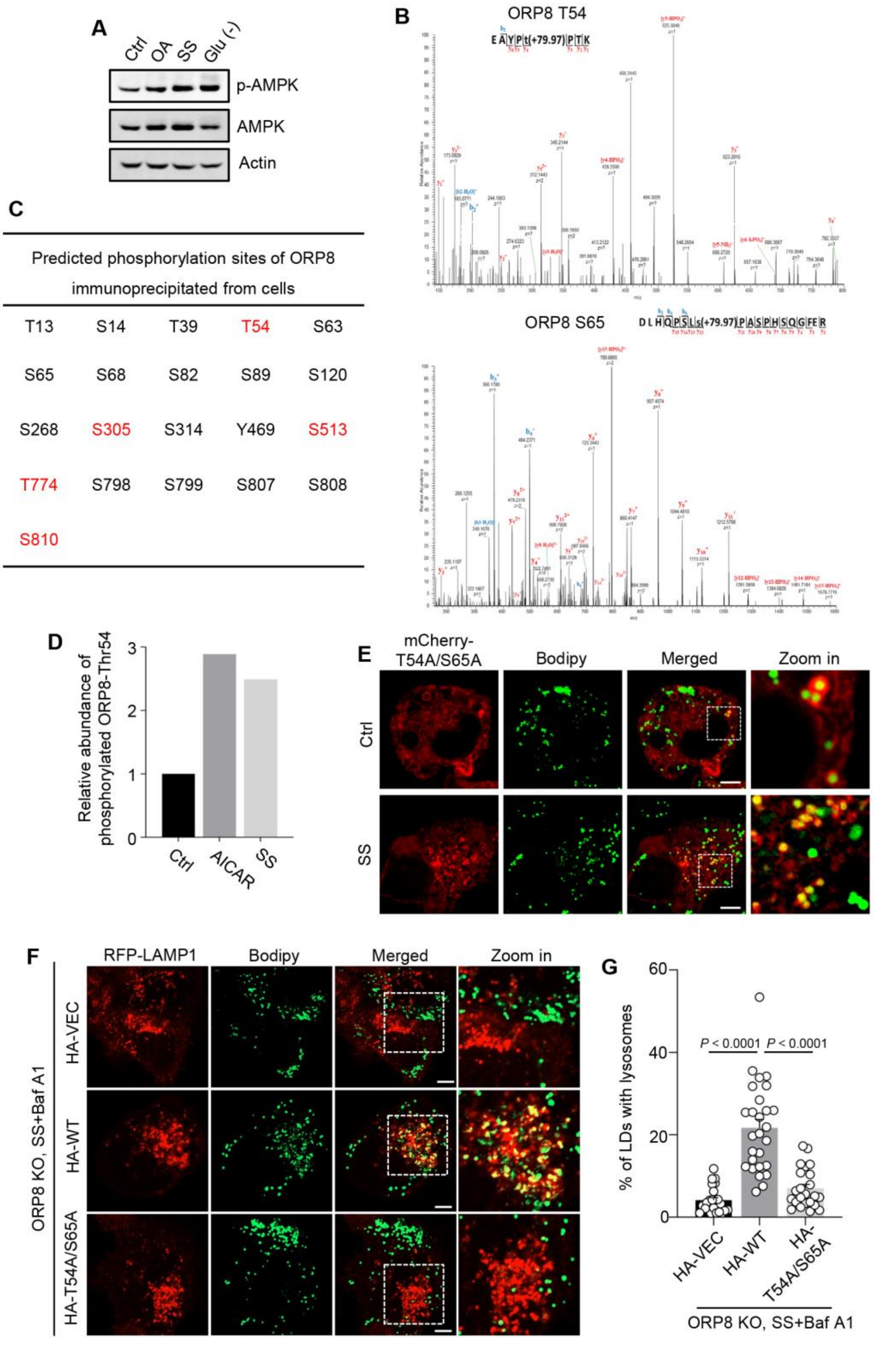

Figure S5. AMPK phosphorylates ORP8 at T54 and S65.

(A) HeLa cells were glucose-starved for 4 h, or serum-starved for 24 h, or treated with 200 μM OA for 6 h. Cellular proteins were analyzed by Western blot with AMPK and p-AMPK antibodies. (B) Mapping phosphorylation site(s) on ORP8 by mass

spectrometry. MS/MS spectrum of 2 tryptic peptides (inset) from kinase-active AMPK complex-incubated recombinant ORP8 protein with a mass shift of 79.97 Da at T54 (left) or S65 (right). (C) Diagram of all the phosphorylation sites of ORP8 identified by LC/MS/MS. HEK293T cells were transfected with HA-ORP8 and treated with or without 2 mM AICAR for 1 h or serum starvation for 24 h. HA-ORP8 was then immunoprecipitated from the cells using an anti-HA antibody and subjected to LC/MS/MS analysis. The residues with increased phosphorylation in both AICAR-treated and serum-starved cells were highlighted in red. (D) Relative abundance of phosphorylation of ORP8 Thr54 identified by LC/MS/MS. The stoichiometric ratio of phosphorylation at Thr54 was shown in (Table S2). (E) Live-cell images of HeLa cells transiently expressing mCherry-ORP8 T54A/S65A. Cells were treated with or without serum starvation for 24 h and stained by Bodipy. (F) Confocal images of ORP8-KO cells transfected with RFP-LAMP1 and ORP8-WT or ORP8-T54A/S65A. Cells were treated with serum starvation for 24 h and 100 nM bafilomycin A1 for 4 h. (G) Quantification of the percentage of LDs co-localized with lysosomes in (F). n = 23 cells. SS, serum starvation; OA, oleate acid; Glu (-), glucose starvation; Baf A1, bafilomycin A1. Scale bars, 5 μm.

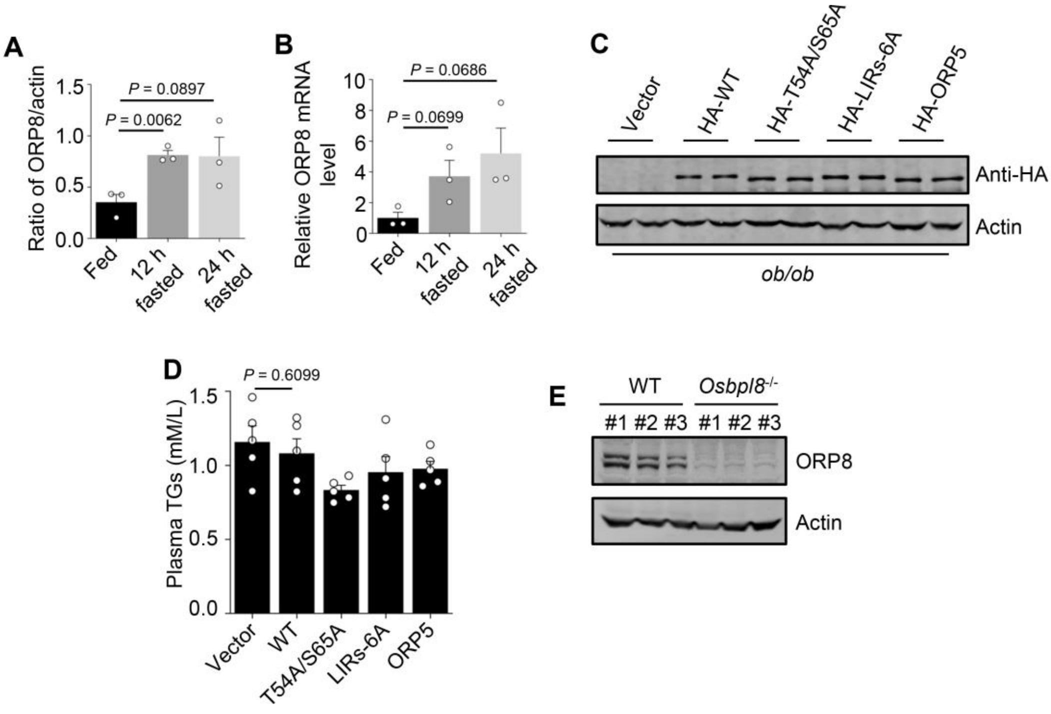

Figure S6. ORP8 reduces LDs and TG in mouse liver.

(A) Quantification of the relative protein level of ORP8 in livers of *ob*/*ob* mice from (Fig. 6A). n = 3 mice. (B) mRNA level of ORP8 in *ob*/*ob* mice liver tissue. n = 3 mice. (C) Expression of HA-tagged ORP8/5 protein in the liver of *ob*/*ob* mice intraperitoneally injected with HA-tagged rAAV-ORP8-WT, rAAV-ORP8-T54A/S65A, rAAV-ORP8-LIRs-6A or rAAV-ORP5. (D) TG levels in the plasma of *ob*/*ob* mice in (C). n = 5 mice. (E) Expression of endogenous ORP8 in the liver of WT and *Osbpl8*$^{-/-}$ mice.
